# Effects of Isomorphous Substitution on Photophysical and Magnetic Properties of Complexes [Ln_1‐_ *_x_* Dy*_x_* (MeDPQ)_2_Cl_3_] (Ln = Y^3+^, Ho^3+^, and Er^3+^)

**DOI:** 10.1002/chem.202502070

**Published:** 2025-10-23

**Authors:** Maksim A. Zhernakov, Yuriy G. Denisenko, Ruslan G. Batulin, Ildar.I. Mirzayanov, Elza D. Sultanova, Maxim S. Molokeev, Vladimir A. Burilov, Valery G. Shtyrlin, Klaus Müller‐Buschbaum

**Affiliations:** ^1^ Institute of Inorganic and Analytical Chemistry Justus‐Liebig‐University Giessen Heinrich‐Buff‐Ring 17 35392 Giessen Germany; ^2^ A. M. Butlerov Chemistry Institute Kazan Federal University Kremlevskaya St., 18 Kazan 420008 Russia; ^3^ School of Natural Sciences Tyumen State University Tyumen 625003 Russia; ^4^ Department of Construction Materials Industrial University of Tyumen Tyumen 625000 Russia; ^5^ Institute of Physics Kazan Federal University Kremlevskaya St., 18 Kazan 420008 Russia; ^6^ Department of Analytical Chemistry Certification and Quality Management Institute of Petroleum Chemistry and Nanotechnologies Kazan National Research Technological University Karl Marx St., 68 Kazan 420015 Russia; ^7^ Laboratory of Crystal Physics Kirensky Institute of Physics Federal Research Center KSC SB RAS Krasnoyarsk 660036 Russia; ^8^ Department of Engineering Physics and Radioelectronic Siberian Federal University Krasnoyarsk 660041 Russia; ^9^ Department of Physics Far Eastern State Transport University Serysheva St. 47 Khabarovsk 680021 Russia; ^10^ Center for Materials Research (LaMa) Justus‐Liebig‐University Giessen Heinrich‐Buff‐Ring 16 35392 Giessen Germany

**Keywords:** exciplex, magnetic interactions, NIR emission, re‐absorption, single‐ion anisotropy

## Abstract

This work encompasses the study of magnetic, optical, and structural properties of the coordination compounds [Ln(MeDPQ)_2_Cl_3_] (Ln ─ Ho^3+^, Er^3+^, Dy^3+^, and Y^3+^; MeDPQ − 2‐methyldipyrido‐[3,2‐f:2′,3′‐h]‐quinoxaline) and substituted complexes [Ln_1‐_ *_x_* Dy*_x_* (MeDPQ)_2_Cl_3_] (Ln = Ho^3+^, Er^3+^, and Y^3+^) based on them. Magnetic measurements within the range 5–300 K revealed single ion anisotropy in [Dy(MeDPQ)_2_Cl_3_], with the Curie‐Weiss temperature *θ* being −3.69 ± 0.03 K. Complexes of Ho^3+^ and Er^3+^ exhibited *f*–*f* emission in the visible range, while the latter was also emissive in the NIR. Dilution of the Dy^3+^ complex with diamagnetic Y^3+^ ions resulted in alterations of magnetic and photophysical properties. The substituted complexes Y_0.5_Dy_0.5_ and Y_0.9_Dy_0.1_ demonstrated paramagnetic behavior, with *θ* being 3.06 ± 0.12 K and 9.64 ± 0.23 K, respectively. In both cases, the emission decay times of Dy^3+^ changed insignificantly, 21.02 ± 0.41 µs and 14.56 ± 0.22 µs, respectively, compared to the value (18.92 ± 0.03 µs) of the individual Dy^3+^ complex. Additional ligand‐based emission bands were observed in the Ho^3+^ and Er^3+^ complexes at room temperature and 77 K and in the substituted complexes Ho_0.5_Dy_0.5_ and Er_0.5_Dy_0.5_ at room temperature, which were assigned to the exciplex states. The thermal stability of [Er_0.5_Dy_0.5_(MeDPQ)_2_Cl_3_] was determined to be the same as for the individual complexes, starting to oxidize at 410°C.

## Graphical abstract

We present the research on the coordination compounds [Ln(MeDPQ)_2_Cl_3_] with Dy^3+^, Ho^3+^, and Er^3+^ ions and the solid solutions [Ln_1‐_ *_x_* Dy*_x_* (MeDPQ)_2_Cl_3_] based on them. The investigated materials exhibited multifunctionality: they absorb light in the UV, visible, and NIR range; emit in the visible and NIR range; demonstrate weak magnetic interactions; and possess exceptional thermal stability.

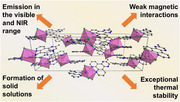

## 1 Introduction

Peculiarities of lanthanides (Ln) are still encouraging scientists to examine temperature‐dependent luminescence, ^[^ ^1^, ^2^ ^]^ magnetic properties, ^[^ ^3^, ^4^, ^5^, ^6^ ^]^ properties of nonlinear optics, ^[^ ^7^ ^]^ and negative thermal expansion. ^[^ ^8^, ^9^ ^]^ Nonetheless, the properties of Ln‐containing compounds are not limited to those already described, as a combination of them within a single phase is possible. In particular, numerous works are being published regarding coordination compounds, coordination polymers, and MOFs possessing both temperature‐dependent luminescence and the behavior of a single‐molecule magnet (SMM). ^[^ ^10^, ^11^ ^]^

In this regard, trivalent dysprosium and its compounds are of high demand since nine electrons on its 4*f* subshell cause the largest total angular momentum *J* among all Ln^3+^ ions and the largest *χT* value of 14.17 cm^3^mol^−1 ^K at 298 K. ^[^ ^12^ ^]^ Moreover, eye‐visible luminescence of the Dy^3+^ compounds can be observed in several cases. ^[^ ^2^, ^13^, ^14^ ^]^ Achieving a high anisotropy of the Dy^3+^ environment requires different ligands in equatorial and axial positions; thus, a usual choice is weaker donors at an equator and stronger donors at axes. ^[^ ^5^, ^15^ ^]^ For instance, N,N’‐*bis*(2‐methylenepyridinyl)ethylenediamine (bpen) or N,N’‐bis(2‐hydroxybenzyl)‐N,N’‐*bis*(2‐methylpyridyl)ethylenediamine (bbpen) and aryloxide or alkoxide ligands are such weaker and stronger donors, respectively.

Up to date, these multifunctional materials usually bear only the Dy^3+^ ion, which operates as an emission and magnetic center. ^[^ ^10^, ^11^, ^16^ ^]^ Therefore, a relatively low quantum yield (rarely exceeding 10%) and the requirement for calibration lead to limited applications of these materials. To this end, implementation of the second Ln^3+^ ion by isomorphous substitution is helpful, as this eliminates calibration requirements ^[^ ^17^ ^]^ and can improve quantum yield. ^[^ ^2^ ^]^ Thus, structural analysis is the first and most helpful technique to prove structural compliance between individual complexes and the resulting solid solution.

The proven complexes of Ln^3+^ ions with 2‐methyldipyrido‐[3,2‐f:2′,3′‐h]‐quinoxaline (MeDPQ), which were already investigated with structural methods ^[^ ^2^, ^18^ ^]^ and showed isotypicity, stability under synthetic air atmosphere to at least 390°C, and temperature‐dependent luminescent properties, ^[^ ^2^ ^]^ are a perfect starting point to discover multifunctional materials. To this end, the constitution [Ln(MeDPQ)_2_Cl_3_] was used to study the new complexes with Ho^3+^ and Er^3+^ ions and substituted complexes [Ln_1‐_ *_x_* Dy*_x_* (MeDPQ)_2_Cl_3_] (Ln = Ho^3+^, Er^3+^, and Y^3+^) based on them by structural, magnetic, and optical methods and simultaneous thermal analysis.

## 2 Results and Discussion

### 2.1 Synthesis and Structure Analysis

**Figure 1:**
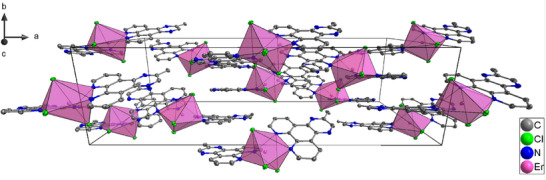
Unit cell of the compound [Er(MeDPQ)_2_Cl_3_]. Thermal ellipsoids describe a 50% probability level of atoms; hydrogen atoms are omitted for clarity: C–gray, Cl–green, N–blue, and Er–pink. Polyhedra represent a coordination environment around the Er atom. The view is along *c* axis.

**Figure 2:**
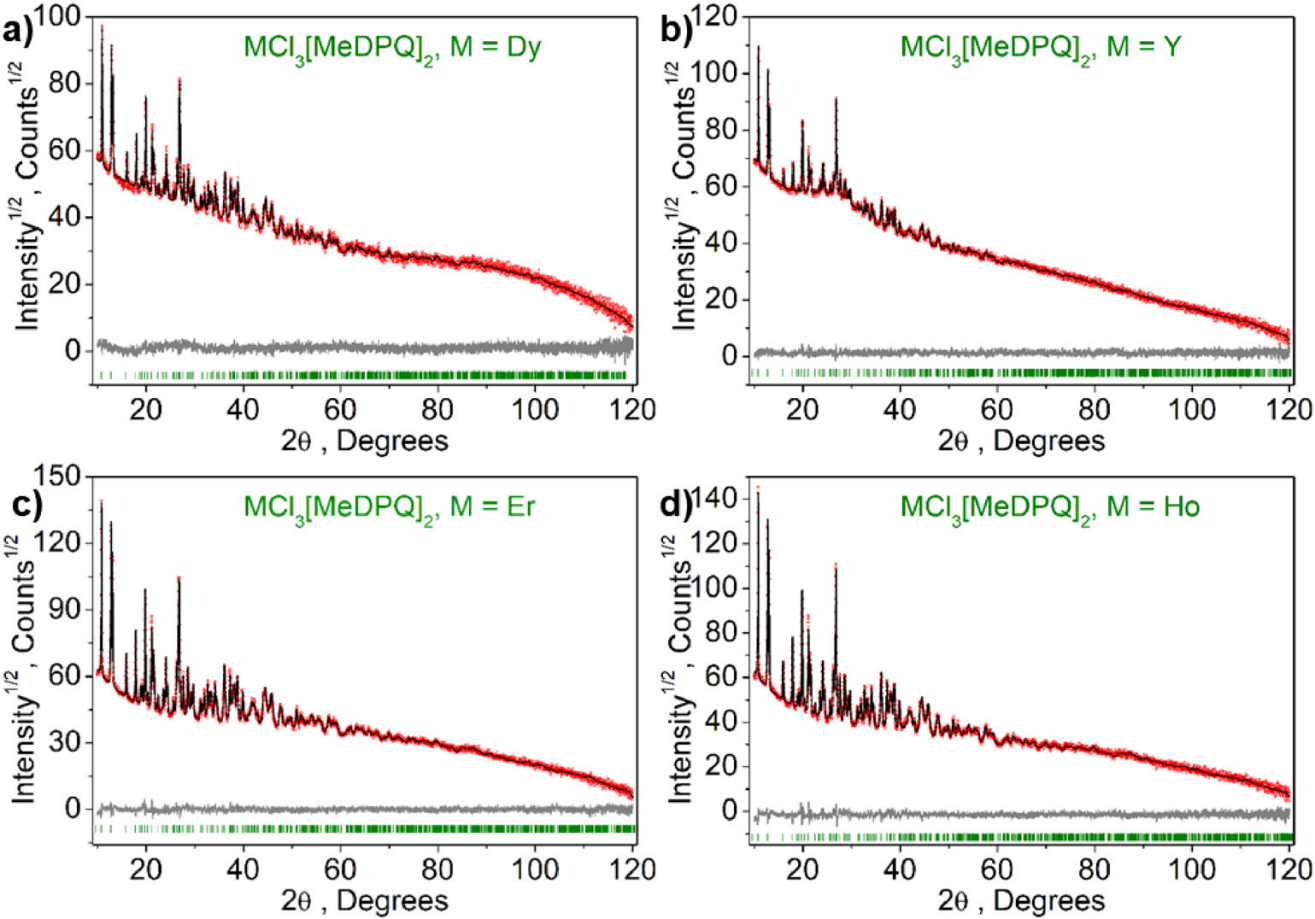
Difference Rietveld plot of the complexes [Ln(MeDPQ)_2_Cl_3_]: **a)** Ln = Dy^3+^; **b)** Ln = Y^3+^; **c)** Ln = Er^3+^; **d)** Ln = Ho^3+^.

**Figure 3:**
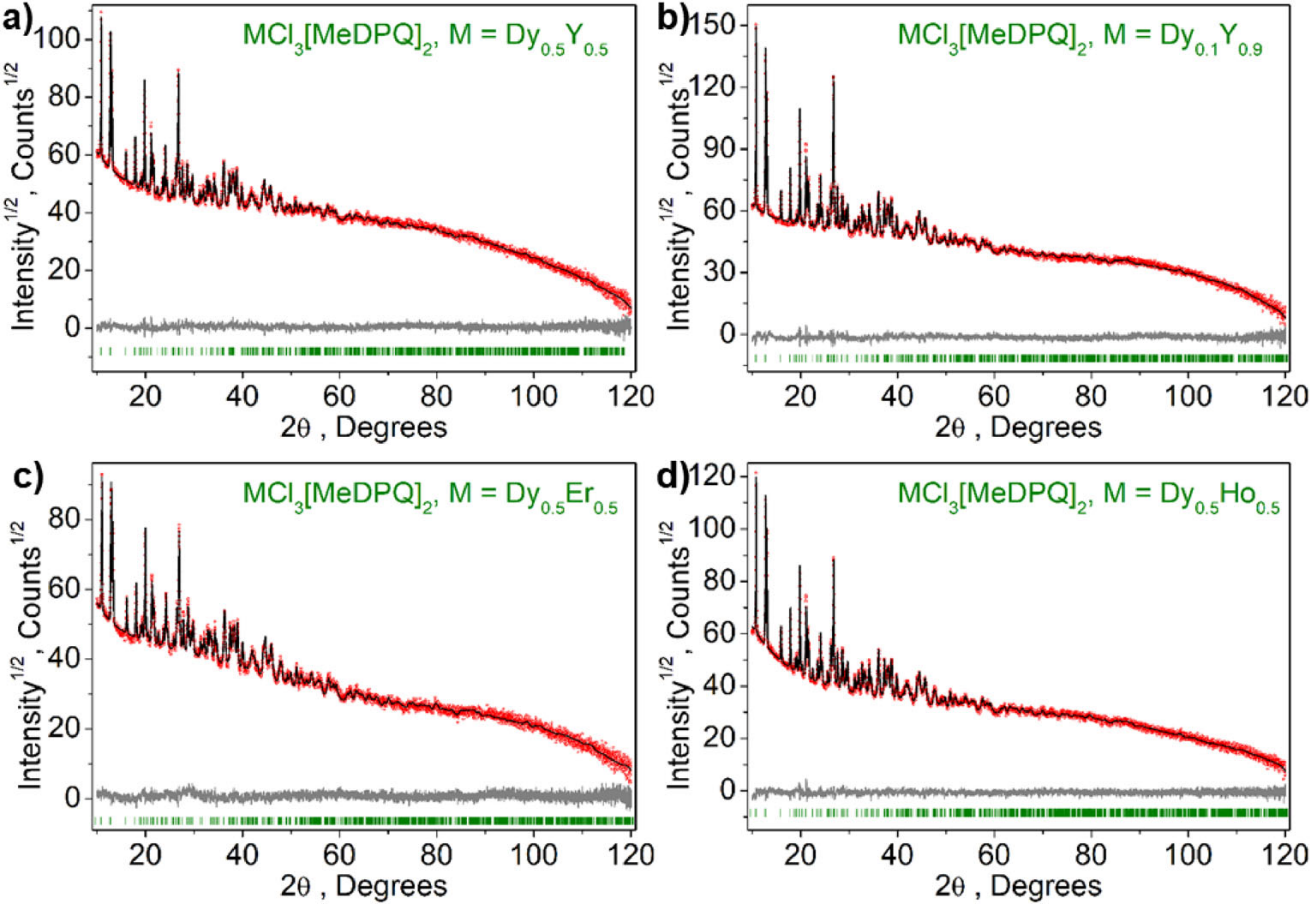
Difference Rietveld plot of the substituted complexes [Ln_1‐_ *_x_* Dy*_x_* (MeDPQ)_2_Cl_3_]: **a)** Y_0.5_Dy_0.5_; **b)** Y_0.9_Dy_0.1_; **c)** Dy_0.5_Er_0.5_; **d)** Dy_0.5_Ho_0.5_.

All complexes reported were synthesized by a reaction of the corresponding salt LnCl_3_∙6H_2_O and the ligand MeDPQ in pyridine at 125°C with high yields (>85%). These compounds crystallize in the orthorhombic space group *Fdd*2, having a central atom with a coordination number of seven. The central atom is surrounded by three chlorine ions and two ligand molecules; thus, the coordination environment forms a distorted pentagonal bipyramid (Figure 1). The detailed crystal data and refinement parameters are reported in Table S1. The complexes [Ln(MeDPQ)_2_Cl_3_] (Ln = Dy^3+^, Y^3+^, Ho^3+^, Er^3+^) and their substituted products [Y_0.5_Dy_0.5_(MeDPQ)_2_Cl_3_] (Y_0.5_Dy_0.5_), [Y_0.9_Dy_0.1_(MeDPQ)_2_Cl_3_] (Y_0.9_Dy_0.1_), [Dy_0.5_Ho_0.5_(MeDPQ)_2_Cl_3_] (Dy_0.5_Ho_0.5_), and [Dy_0.5_Er_0.5_(MeDPQ)_2_Cl_3_] (Dy_0.5_Ho_0.5_) are isotypical with the previously described compound [Yb(MeDPQ)_2_Cl_3_], ^[^ ^18^ ^]^ which was confirmed by powder X‐ray diffraction, Rietveld refinement (Figures 2, 3), and vibrational spectroscopy (Figure S1).

In order to obtain unit cell parameters of better quality, we applied Rietveld refinement for powder diffractograms, which were recorded in the range of 10–120 degrees in 2*θ* at room temperature (Figures 2, 3). The calculated parameters (Tables 1, 2) approved phase purity and isotypicity of mono‐lanthanide and bi‐lanthanide complexes; hence, unobstructed formation of solid solutions can be used to combine diverse physical properties of different Ln^3+^ ions.

**Table 1:** Main parameters of processing and refinement of the individual Dy^3+^ and Y^3+^ compounds and substituted complexes at room temperature

Compound	Space Group	Cell parameters [Å], Cell Volume [Å^3^]	*R*_wp_	*R*_p_	*R*_B_	*χ* ^2^
[Dy(MeDPQ)_2_Cl_3_]	*Fdd2*	*a* = 37.178(4), *b* = 10.3313(9), *c* = 14.6280(14), *V* = 5618.6(9)	5.13	3.75	1.42	1.81
[Y(MeDPQ)_2_Cl_3_]		*a* = 37.158(5), *b* = 10.3368(12), *c* = 14.620(2), *V* = 5615.5(13)	3.60	2.46	0.91	1.40
[Y_0.5_Dy_0.5_(MeDPQ)_2_Cl_3_]		*a* = 37.193(2), *b* = 10.3401(6), *c* = 14.6273(12), *V* = 5625.3(7)	4.40	3.16	1.21	1.72
[Y_0.9_Dy_0.1_(MeDPQ)_2_Cl_3_]		*a* = 37.183(2), *b* = 10.3418(5), *c* = 14.6267(9), *V* = 5624.5(6)	4.72	3.46	1.42	2.10

**Table 2:** Main parameters of processing and refinement of the individual Ho^3+^ and Er^3+^ compounds and substituted complexes with Dy^3+^ ion at room temperature

Compound	Space Group	Cell parameters [Å], Cell Volume [Å^3^]	*R*_wp_	*R*_p_	*R*_B_	*χ* ^2^
[Ho(MeDPQ)_2_Cl_3_]	*Fdd2*	*a* = 37.136(2), *b* = 10.3328(5), *c* = 14.6271(8), *V* = 5612.6(5)	5.30	3.86	1.54	1.94
[Er(MeDPQ)_2_Cl_3_]		*a* = 37.163(2), *b* = 10.3456(5), *c* = 14.6217(7), *V* = 5621.7(5)	4.34	3.19	1.27	1.67
[Er_0.5_Dy_0.5_(MeDPQ)_2_Cl_3_]		*a* = 37.171(4), *b* = 10.332(1), *c* = 14.614(2), *V* = 5613(1)	5.42	3.93	1.89	1.86
[Ho_0.5_Dy_0.5_(MeDPQ)_2_Cl_3_]		*a* = 37.169(3), *b* = 10.3356(7), *c* = 14.6350(11), *V* = 5622.3(7)	4.71	3.49	1.23	1.64

Tables 1, 2 present the unit cell parameters of the synthesized materials. According to the decrease of the crystal radius value in the series Dy^3+^ (1.11 Å)→Ho^3+^ (1.10 Å)→Y^3+^ (1.10 Å)→Er^3+^ (1.085 Å), ^[^ ^19^, ^20^ ^]^ decreasing values of the parameters were expected as well. A similar tendency is observed in the series of cell volumes for the Dy^3+^, Ho^3+^, and Y^3+^ complexes, since the values at room temperature are 5618.6(9) Å^3^, 5615.5(13) Å^3^, and 5612.6(5) Å^3^, respectively. However, the volume of the Er^3+^ complex is slightly out of the sequence, having a value of 5621.7(5) Å^3^.

Analysis of the parameters of the substituted complexes is even more challenging compared to those for the individual complexes. For instance, it was expected that values of the cell volume in the case of the Y_0.9_Dy_0.1_ and Y_0.5_Dy_0.5_ solid solutions would be considerably different, but there is little difference within two absolute standard deviation intervals (5624.5(6) Å^3^ and 5625.3(7) Å^3^, respectively). Furthermore, the cell volumes of substituted complexes are even larger than those of the individual Dy^3+^ complex (5624.51(56) Å^3^ and 5618.64(90) Å^3^, respectively). These results make the structural analysis of coordination compounds demand additional data because their lattices are not densely packed. Shannon radii are widely used to explain several effects on structure motives in series of lanthanide compounds, ^[^ ^13^, ^21^, ^22^ ^]^ despite the fact that they were determined for lanthanide oxides and fluorides, which are densely packed. Hence, these radii can only be used for qualitative purposes in the case of coordination compounds.

In our case of the large orthorhombic unit cell, slightly different packing of the molecular units is very likely to occur. Therefore, the different packing can lead to close unit cell parameters for various Ln^3+^ ions. However, if such alterations proceed without the addition of new symmetry elements, it will be impossible to spot this on a powder diffractogram. Consequently, a substance preserves the same pattern regardless of packing options. That was previously observed in substituted complexes of Sm^3+^, Eu^3+^, Gd^3+^, and Tb^3+^ based on coordination compounds. ^[^ ^2^ ^]^ In addition to alterations in the unit cell parameters, we have confirmed the presence of the second Ln^3+^ by magnetic and optical measurements.

The IR spectra of the synthesized compounds demonstrate the same vibrational modes in each case. And the vibrational modes of the ligand MeDPQ completely determine spectra patterns (Figure S1). In particular, the vibrations of the coordinated nitrogen atoms are shifted toward lower wavenumbers compared to the spectra of the individual ligand. ^[^ ^2^ ^]^ Furthermore, fewer bands are observed in the region of 1000–400 cm^−^^1^, evidencing rigid coordination bonds between the lanthanide and nitrogen atoms. The latter results in reduced mobility of the atoms along the C─N and C═N bonds, while vibrational modes of the rest moiety do not undergo significant transformations.

### 2.2 Magnetic Properties

**Figure 4:**
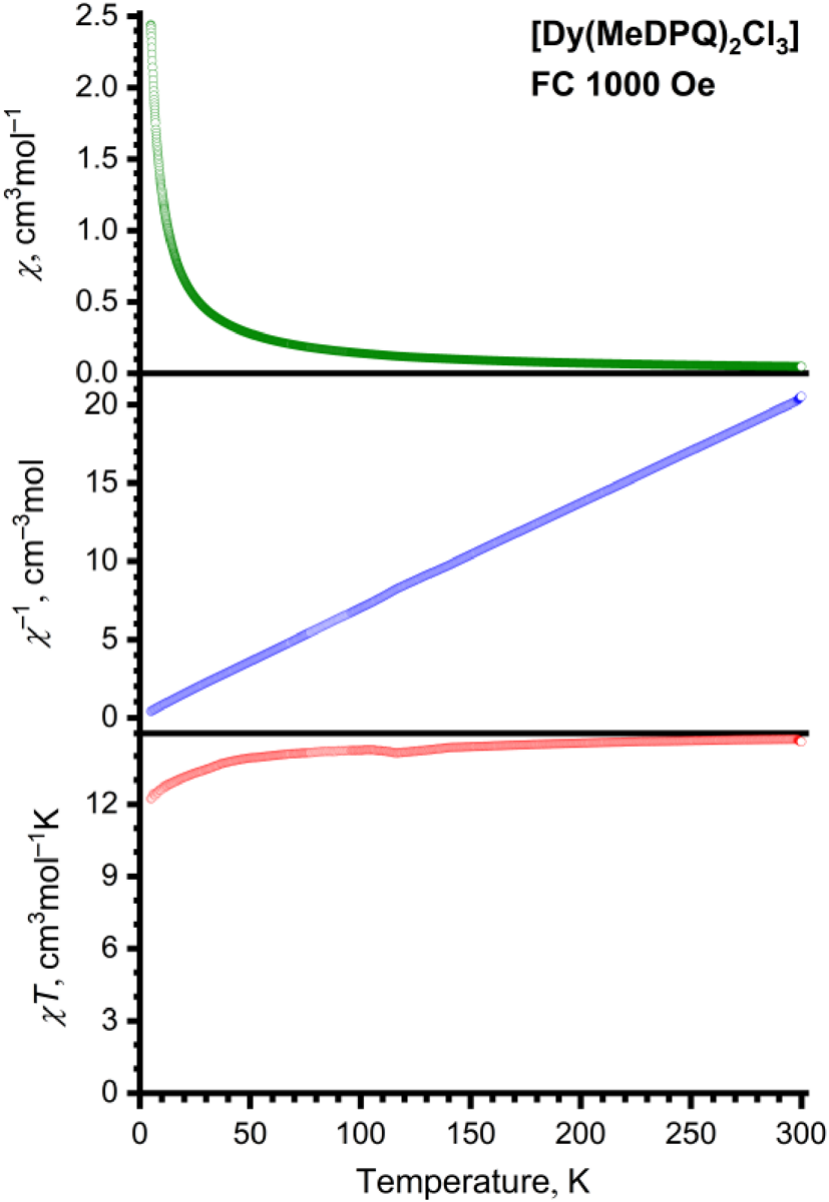
Temperature dependence of the magnetic susceptibility of the solid‐state complex [Dy(MeDPQ)_2_Cl_3_] measured at 1000 Oe and FC regime within the temperature range of 5–300 K.

Magnetic measurements were performed using a vibrating sample magnetometer (VSM option) of the Physical Property Measurement System (PPMS‐9, Quantum Design, USA) in zero‐field‐cooled (ZFC) and field‐cooled (FC) regimes at magnetic fields of 100 and 1000 Oe, within the temperature range of 5–300 K. Figure 4 presents the temperature dependence of magnetic susceptibility *χ* for the solid complex [Dy(MeDPQ)_2_Cl_3_] along with reverse magnetic susceptibility *χ* ^−^^1^ and *χT* product.

Since magnetic susceptibility *χ* of the Ln^3+^ ions obeys the Curie‐Weiss law only in the case of free ion approximation, that is, neglecting ligand field, spin‐orbit coupling, and thermal depopulation, a modified version of the law (Equation 1) has to be used. ^[^ ^12^, ^23^, ^24^ ^]^

(1) $$\chi =\frac{C}{T-\theta }+\chi_{0}$$

where *C* is the Curie constant, *T* is absolute temperature, *χ*_0_ is temperature‐independent magnetic susceptibility, and *θ* is Curie‐Weiss temperature. To check if this is the case, the experimental data was analyzed for the deviation from a linear trend using a *χ*−^1^ versus *T* plot (Figure S2). Since the curvature of the plot was barely observed, the Curie‐Weiss law (Equation 2) was applied without a temperature‐independent contribution to magnetic susceptibility *χ*_0_. ^[^ ^23^ ^]^

(2) $$\chi =\frac{C}{T-\theta }\;\Rightarrow \;\;\chi^{-1}=\frac{T}{C}-\frac{\theta }{C}$$

The resulting fit yielded a Curie‐Weiss temperature *θ* of −3.69 ± 0.03 K for [Dy(MeDPQ)_2_Cl_3_]. Figure 4 summarizes the *χ* versus *T*, *χ* ^−^^1^ versus *T*, and *χT* versus *T* plots. Referring to the latter parameter, the *χT* value at 298 K is 14.7 cm^3^mol^−^^1 ^K, which is close to the theoretical *χT* value of 14.17 cm^3^mol^−^^1 ^K for the free ion approximation. ^[^ ^12^, ^23^ ^]^ The *χT* value slowly decreases upon cooling to 50 K, where it starts to decrease rapidly, eventually reaching the value of 12.2 cm^3^mol^−^^1 ^K at 5 K. This magnetic behavior is considered typical for Dy^3+^ complexes, as at lower temperatures both thermal depopulation of *m*_J_ states and magnetic interactions are possible. ^[^ ^4^, ^5^, ^23^ ^]^ Similar features were observed for several Dy^3+^ coordination compounds with O–donors ^[^ ^16^, ^25^, ^26^, ^27^ ^]^ and Dy^3+^ complex with the constitution ^2^ **_∞_** [Ln_2_(3‐PyPzH)_3_Cl_6_]·2MeCN, 3‐PyPzH − 3‐(3‐pyridyl)pyrazole. ^[^ ^4^ ^]^

**Figure 5:**
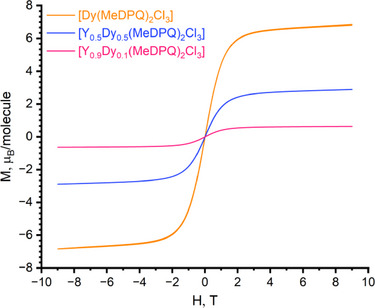
Field‐dependent measurements of magnetic moment for the solid‐state compounds [Dy(MeDPQ)_2_Cl_3_], [Y_0.5_Dy_0.5_(MeDPQ)_2_Cl_3_], and [Y_0.9_Dy_0.1_(MeDPQ)_2_Cl_3_] performed at 5 K.

Figure 5 demonstrates field‐dependent measurements of magnetization at 5 K, where hysteresis is almost absent. This dependency allowed us to determine the magnetic moment corresponding to the molecule [Dy(MeDPQ)_2_Cl_3_], which has a value of 6.8 *µ*_B_. The resulting value differs from the theoretically calculated effective magnetic moment *µ*_eff_ with Equation 3:

(3) $$\mu_{cal}=g_{J}\;\sqrt{J\left(J+1\right)}\mu_{B}$$

where *g_J_* is g‐tensor and *J* is the total angular momentum. For Dy^3+^ ion it gives the value of 10.65 µ_B_ in the case of the free ion approximation. This observation of the unsaturated magnetic state, together with the negative Curie temperature, corresponds to the strong single‐ion anisotropy in the reported compound. For a deeper insight, we have synthesized magnetically diluted samples with the Y^3+^ ions, namely substituted complexes [Y_0.5_Dy_0.5_(MeDPQ)_2_Cl_3_] and [Y_0.9_Dy_0.1_(MeDPQ)_2_Cl_3_]. The complexes [Dy(MeDPQ)_2_Cl_3_] and [Y(MeDPQ)_2_Cl_3_] are isotypic according to PXRD, Rietveld refinement (Figures 2, 3), and vibrational spectroscopy (Figure S1), and the structural description of the solid solutions Y_0.5_Dy_0.5_ and Y_0.9_Dy_0.1_ is presented in the **Synthesis and Structural Analysis** section.

Figures S3, S4 depict the magnetic susceptibility plots against temperature of the diluted samples Y_0.5_Dy_0.5_ and Y_0.9_Dy_0.1_. A closer examination of the *χT* versus *T* plots reveals that the parameter values at 298 K are 7.03 cm^3^mol^−^^1 ^K and 1.40 cm^3^mol^−^^1 ^K for the samples Y_0.5_Dy_0.5_ and Y_0.9_Dy_0.1_, respectively. It is worth noting that these values are approximately 50% and 10% of those for the individual compound [Dy(MeDPQ)_2_Cl_3_], which corroborates the lanthanide content coupled with Rietveld refinement and claimed constitution.

In both cases, the *χT* values remained constant down to 230 K and then slightly increased, reaching local maximal values of 7.41 cm^3^mol^−^^1 ^K and 1.59 cm^3^mol^−^^1 ^K at 121 K for the solid solutions Y_0.5_Dy_0.5_ and Y_0.9_Dy_0.1_, respectively. Afterward, the parameter monotonically decreases, reaching respective *χT* values of 6.33 cm^3^mol^−^^1 ^K and 1.42 cm^3^mol^−^^1 ^K at 5 K. We attribute this behavior to the paramagnetic character of the Dy^3+^ ions within the diluted lattice, which can orient antiparallelly at lower temperatures, resulting in the reduced values of magnetization.

To obtain the main parameters of a magnetic pattern of the substituted complexes Y_0.5_Dy_0.5_ and Y_0.9_Dy_0.1_, the inverse magnetic susceptibility plot *χ* ^−^^1^ versus *T* was approximated with the Curie‐Weiss law (Figures S5, S6). The resulting fit yielded Curie‐Weiss temperature *θ* of 3.06 ± 0.12 K and 9.64 ± 0.23 K and effective magnetic moment *µ*_eff_ of 7.49 ± 0.01 *µ*_B_ and 3.33 ± 0.01 *µ*_B_ for the samples Y_0.5_Dy_0.5_ and Y_0.9_Dy_0.1_, respectively. Hence, the diamagnetically diluted compounds lack interactions between Dy^3+^ ions and exhibit the behavior of a typical paramagnetic. The positive value of Curie temperature *θ* supports this conclusion.

Therefore, the experiments performed demonstrated magnetic unsaturation in the complex [Dy(MeDPQ)_2_Cl_3_] due to single ion anisotropy and weak magnetic interactions between the Dy^3+^ ions. This shall also be valid for other complexes with the constitution [Ln(MeDPQ)_2_Cl_3_], where Ln^3+^ ions with nonzero spin and total angular momentum *J* due to the same crystal structure. This supports our previous findings of the direct interactions between Ln^3+^ ions, which can occur if the distance is below 10 Å, ^[^ ^28^ ^]^ leading to temperature‐dependent luminescent properties. ^[^ ^2^ ^]^

### 2.3 Photophysical Properties

**Figure 6:**
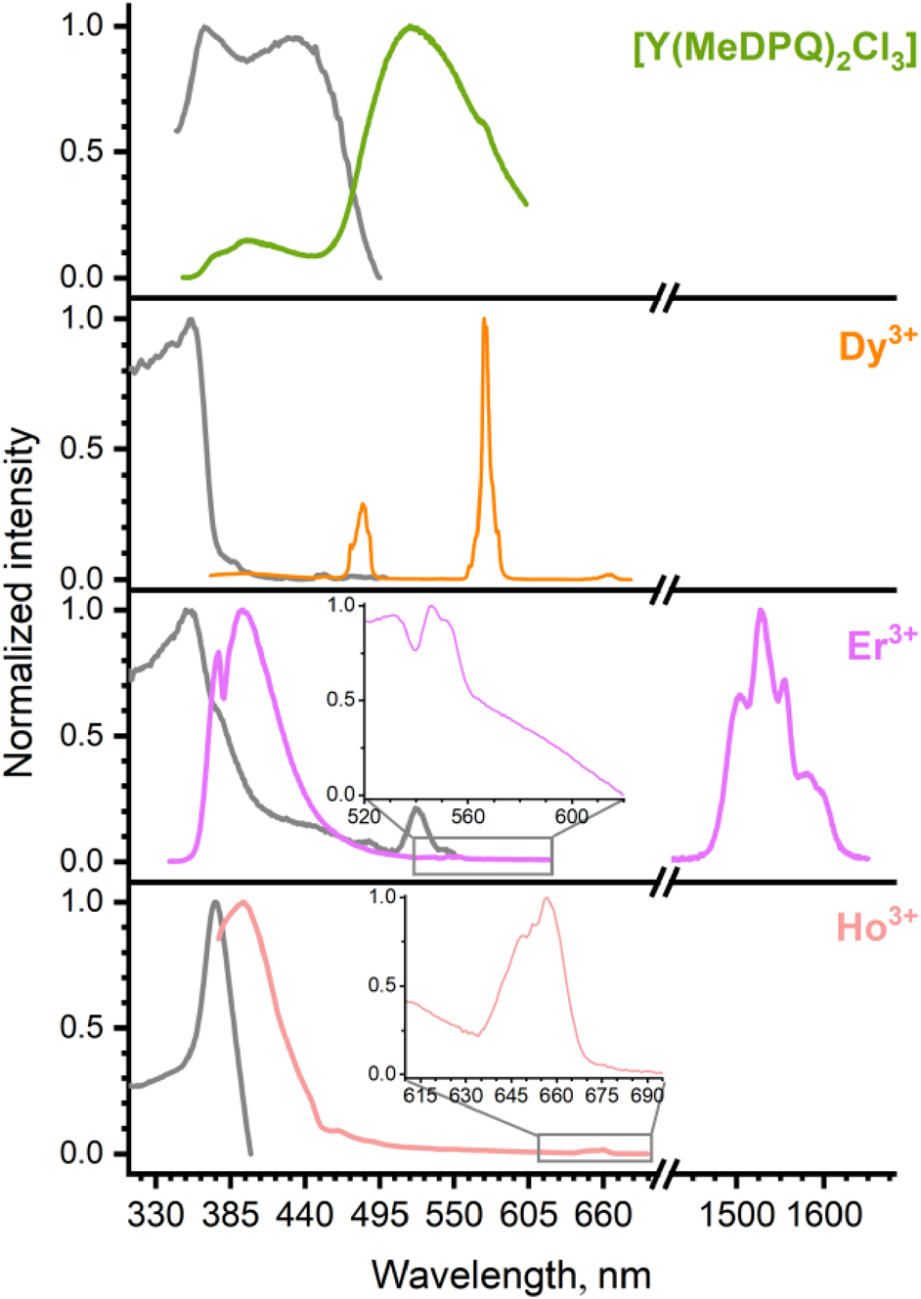
Normalized photoluminescence spectra of the solid‐state complexes [Ln(MeDPQ)_2_Cl_3_] at room temperature. The excitation spectra are presented in gray and were recorded at λ_em_ 517 nm (Y^3+^), 571 nm (Dy^3+^), 1527 nm (Er^3+^), and 420 nm (Ho^3+^), respectively. The emission spectra were recorded at λ_ex_ 325 nm (Y^3+^ complex) and 356 nm (Dy^3+^, Er^3+^ (inset and NIR), and Ho^3+^ complexes). In the case of the Er^3+^ complex, the emission spectrum in the visible range was recorded at λ_ex_ 320 nm. The visible and NIR parts of the Er^3+^ spectrum were normalized separately. The inset spectrum for the Ho^3+^ compound was recorded with a 395 nm cut‐off filter.

To study the photophysical properties of the materials reported, both photoluminescence and diffuse reflectance spectroscopy were used. Figure 6 represents the excitation and emission spectra of the individual complexes [Ln(MeDPQ)_2_Cl_3_] (Ln = Y^3+^, Dy^3+^, Ho^3+^, and Er^3+^).

All complexes exhibit broad‐band ligand‐based emission, which is better observed in the case of the Y^3+^ complex. The emission band in the Y^3+^ complex corresponds to phosphorescence since a large Stokes shift between excitation and emission maxima and a long decay time (τ_em_ = 7.87(5) ms ^[^ ^2^ ^]^) were observed. In the case of [Dy(MeDPQ)_2_Cl_3_], *f–f* emission of this ion almost completely suppresses ligand‐based emission, making the latter barely noticeable. This situation occurs if efficient energy transfer from the ligand to the Ln^3+^ ion proceeds. To this end, the energy difference between a ligand triplet state T_1_ and an excited state of a Ln^3+^ ion shall be approximately 3000 cm^−1^.^[^ ^29^ ^]^ This phenomenon was observed in related systems with N‐donor and Cl ligands. ^[^ ^2^, ^13^, ^14^, ^30^ ^]^

The compound [Er(MeDPQ)_2_Cl_3_] exhibited the characteristic *f–f* transition ^4^I_13/2_→^4^I_15/2_, resulting in the emission in the NIR range. Besides that, we have observed the reabsorption band ^4^G_11/2_←^4^I_15/2_ at 379 nm and low‐intensity *f–f* emission of the Er^3+^ ion in the visible range. In the case of [Ho(MeDPQ)_2_Cl_3_], low‐intensity *f–f* emission of the Ho^3+^ ion also was observed in the visible range (Figure 6, inset). This is not widespread for coordination compounds of Ho^3+^ and Er^3+^ ions, as they rarely exhibit their *f–f* transitions in the visible range luminescence ^[^ ^13^, ^31^, ^32^, ^33^ ^]^ and inorganic materials containing these ions are more often studied as NIR emitters and materials for second harmonic generation.

The investigations at 77 K revealed that both Er^3+^ and Ho^3+^ compounds are still emissive, although the intensity of the Er^3+^ transition in the visible range became too weak for detection (Figures S7–S13). Detailed figures for these spectra (Figures S7–S17) with deciphered *f–f* transitions of the Ln^3+^ ions are presented in the Supporting Information.

**Figure 7:**
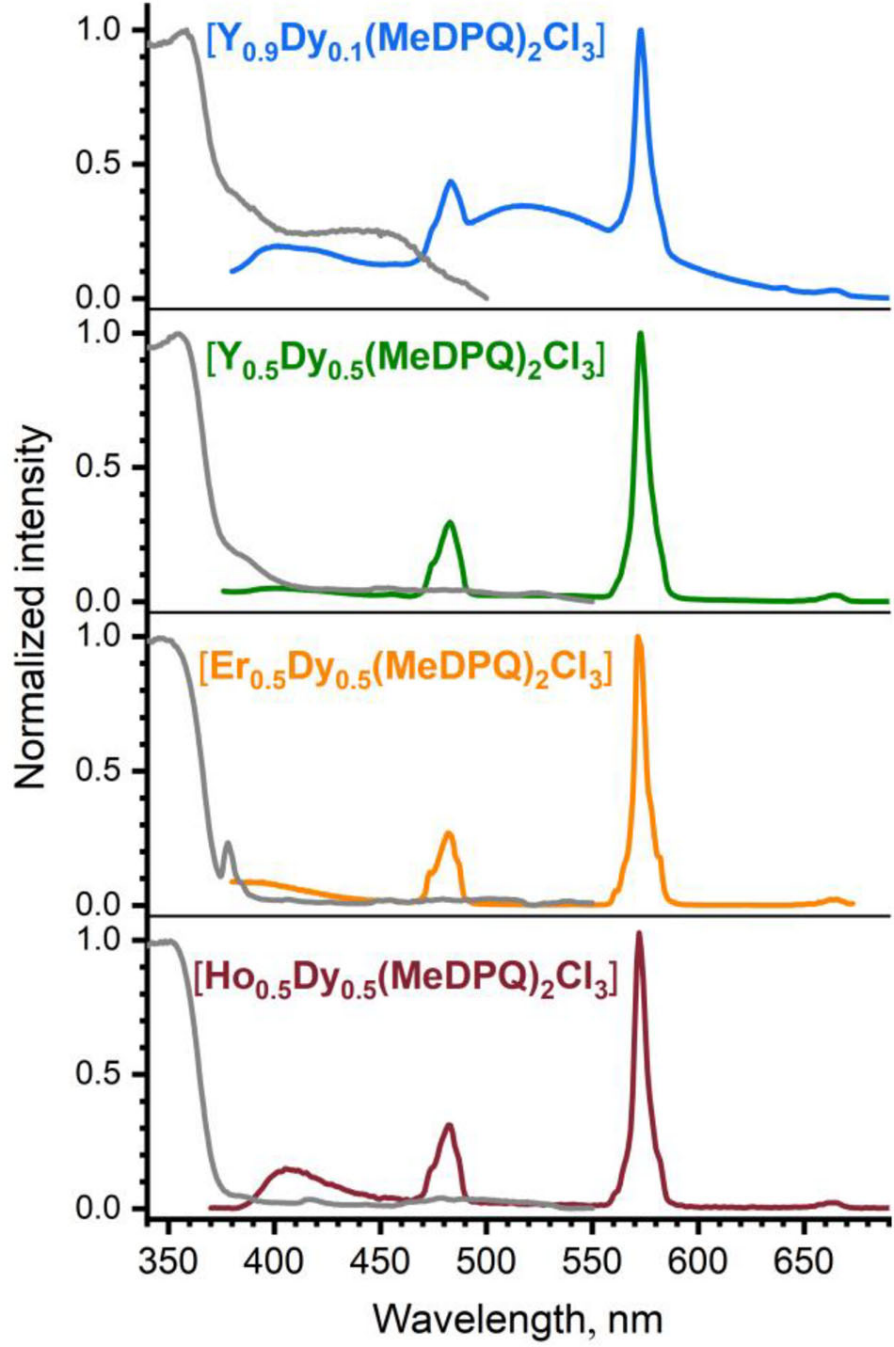
Normalized photoluminescence spectra of the solid‐state substituted complexes [Ln_1‐_ *_x_* Dy*_x_* (MeDPQ)_2_Cl_3_] at room temperature. The excitation spectra are presented in gray and were recorded at λ_em_ 572 nm. The emission spectra were recorded at λ_ex_ 359 nm (Y_0.9_Dy_0.1_), 356 nm (Y_0.5_Dy_0.5_), and 350 nm (Er_0.5_Dy_0.5_ and Ho_0.5_Dy_0.5_).

The excited state ^4^F_9/2_ of the Dy^3+^ ion has the energy of 20 960 cm^−^^1^, which is close to the excited states ^5^F_4_ (18 550 cm^−^^1^) and ^4^S_3/2_ (18 400 cm^−^^1^) of Ho^3+^ and Er^3+^ ions, respectively. Moreover, these ions possess several energy levels that lie below the ^5^F_4_ and ^4^S_3/2_ states, enabling the energy transfer from the ligand MeDPQ and the excited states of Dy^3+^ ion. ^[^ ^34^ ^]^ Hence, bi‐lanthanide systems can be tested for the feasibility of the Dy^3+^→Ho^3+^ and Dy^3+^→Er^3+^ energy transfer. ^[^ ^31^, ^32^, ^35^, ^36^ ^]^ To this end, we studied the photoluminescent properties of the solid solutions [Ln_1‐_ *_x_* Dy*_x_* (MeDPQ)_2_Cl_3_] (Ln = Y^3+^, Ho^3+^, and Er^3+^). Figure 7 presents the excitation and emission spectra of these solid solutions recorded at room temperature. The intensity of the characteristic *f–f* transition ^4^F_9/2_→^6^H_13/2_ (572 nm) of the Dy^3+^ ion remains dominant even in the case of [Y_0.9_Dy_0.1_(MeDPQ)_2_Cl_3_], highlighting the sufficient sensitization of Dy^3+^ ions by the ligand MeDPQ. Furthermore, the quantitative photophysical investigations of [Y_1‐_ *_x_* Dy*_x_* (MeDPQ)_2_Cl_3_] revealed insignificant alterations in the emission decay time of Dy^3+^ ion, compared the value of the individual complex (18.92 ± 0.03 µs ^[^ ^2^ ^]^). The values were calculated using a single exponential fit, yielding decay times of 21.02 ± 0.41 µs and 14.56 ± 0.22 µs for Y_0.5_Dy_0.5_ and Y_0.9_Dy_0.1_, respectively. Thus, we can infer that radiative deactivation through the Dy^3+^ states is a favorable path for this system with the ligand MeDPQ.

**Figure 8:**
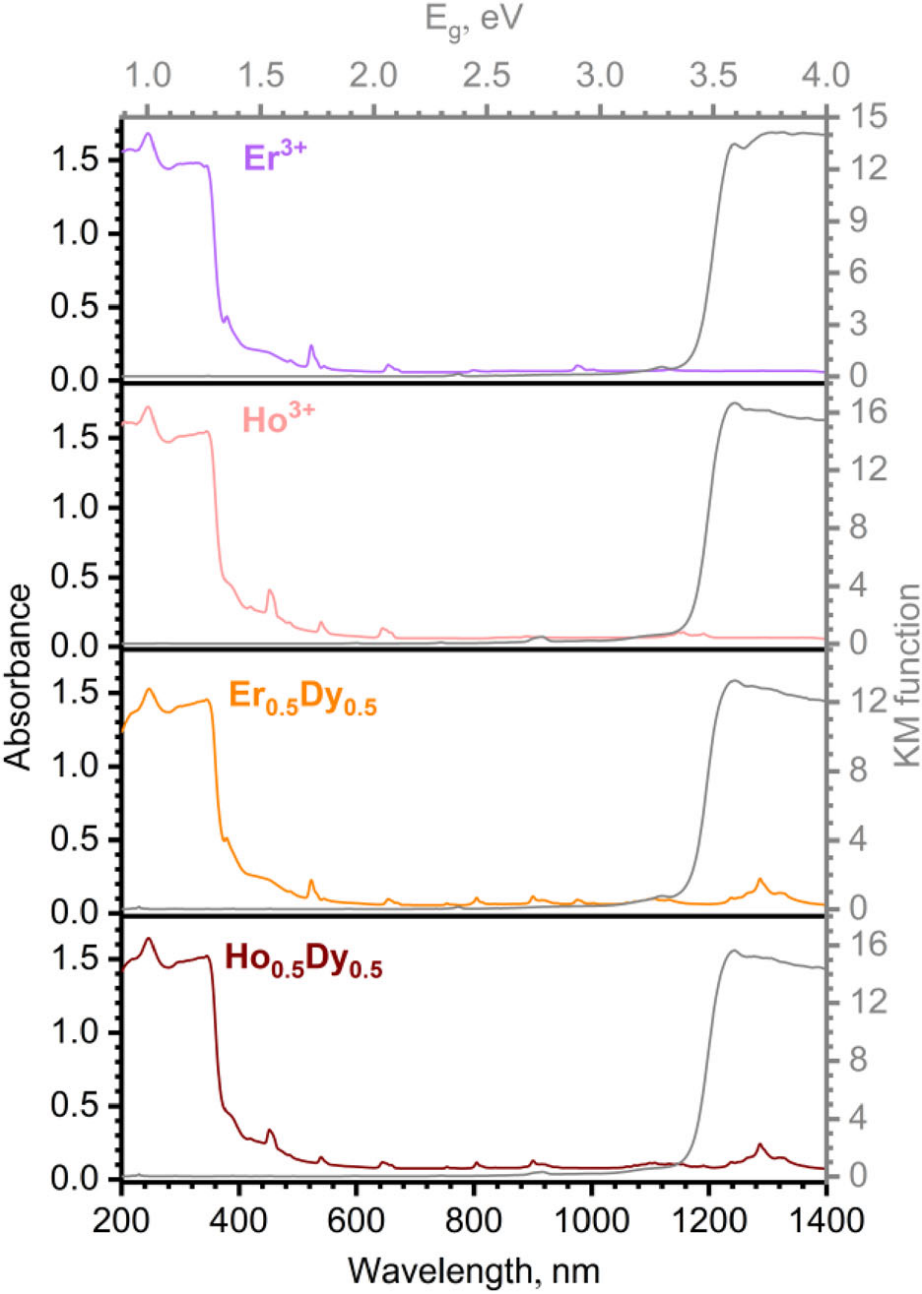
Diffuse reflectance spectra (colored) and Kubelka‐Munk plot (gray) of the solid state Er^3+^ (violet), Ho^3+^ (pink) complexes and Er_0.5_Dy_0.5_ (orange), Ho_0.5_Dy_0.5_ (dark red) substitutional complexes.

Figures 8, S18–S25 represent light absorption profile as absorbance *A* versus wavelength and Kubelka‐Munk function versus energy plots. The latter plot enabled us to determine the band gap value *E_g_*, which was calculated to be in the range of 3.3–3.4 eV. Ln^3+^‐based *f–f* absorption of photons is observed in all cases except for the compound [Y(MeDPQ)_2_Cl_3_] since Y^3+^ is a *d*–element with a filled shell. A set of these *f–f* transitions is responsible for light harvesting in the visible and NIR ranges, while the MeDPQ ligand remains a stronger light absorber in the UV range. Therefore, these compounds can gather light throughout the UV–Vis–NIR range, which subsequently complicates their excitation and emission profiles.

**Figure 9:**
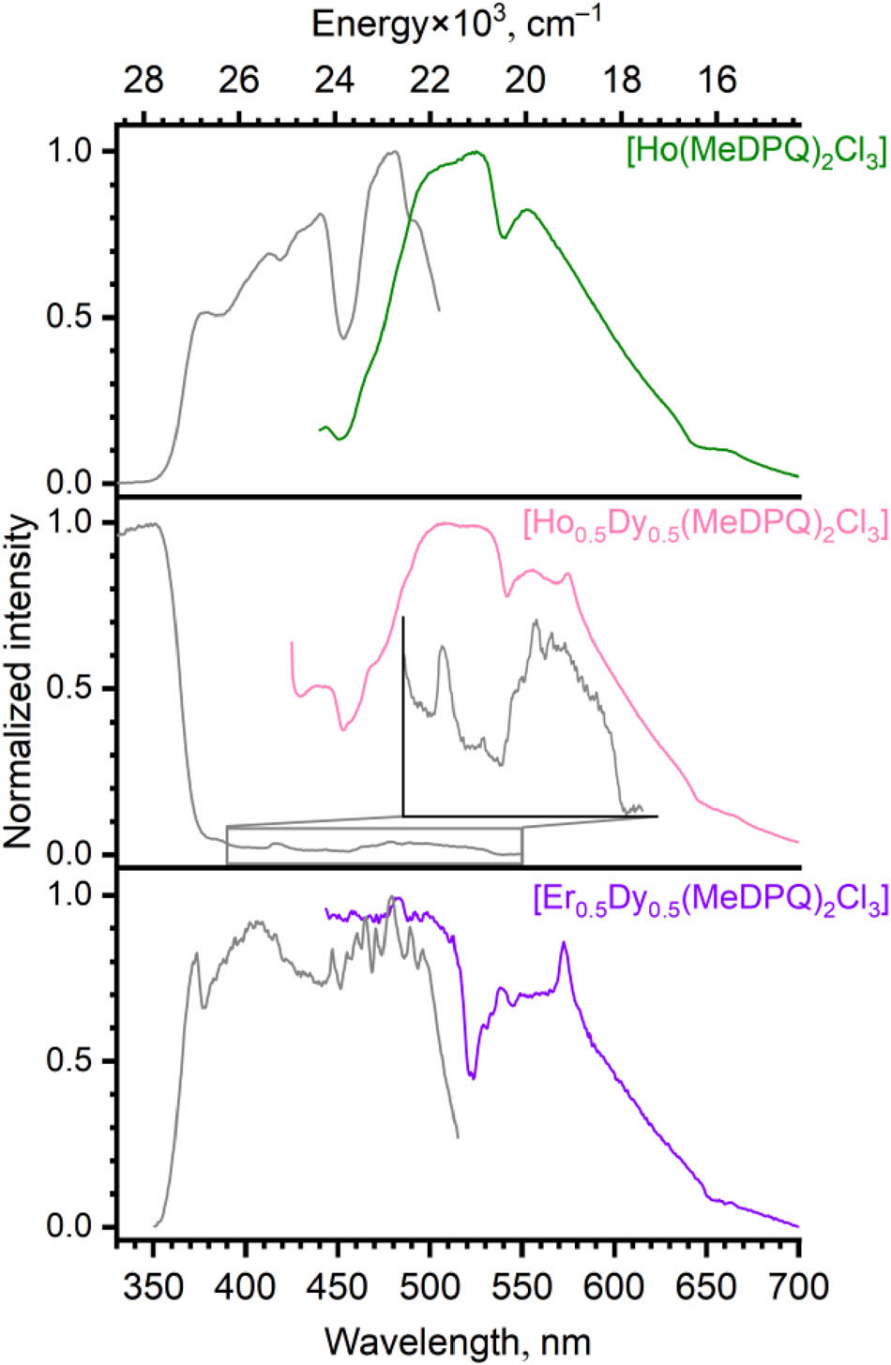
Normalized photoluminescence spectra of the solid‐state complexes [Ho(MeDPQ)_2_Cl_3_] and [Ln_0.5_Dy_0.5_(MeDPQ)_2_Cl_3_] at room temperature, featuring additional emission bands. The excitation spectra are presented in gray and were recorded at λ_em_ 525 nm, 572 nm, and 539 nm for the compounds Ho^3+^, Ho_0.5_Dy_0.5_, and Er_0.5_Dy_0.5_, respectively. The emission spectra were recorded at λ_ex_ 420 nm, 405 nm, and 410 nm for [Ho(MeDPQ)_2_Cl_3_], Ho_0.5_Dy_0.5_, and Er_0.5_Dy_0.5_, respectively.

We have detected additional emission bands in the spectra of [Ho(MeDPQ)_2_Cl_3_] and the solid solutions, Ho_0.5_Dy_0.5_, and Er_0.5_Dy_0.5_ at room temperature (Figure 9) and in the spectra of Er^3+^ and Ho^3+^ complexes at 77 K (Figures S9–S13), which were observed at λ_ex_ > 400 nm. The re‐absorption bands of Ho^3+^ (^5^G_5_; ^5^G_6_; ^5^F_4_←^5^I_8_) and Er^3+^ (^2^H_11/2_; ^4^S_3/2_←^4^I_15/2_) ions are well observed in these spectra, affecting the general mechanism of the energy transfer in the system [Ln(MeDPQ)_2_Cl_3_]. We find noteworthy a recent proposal of the re‐absorption effect in the compound {[Ho^III^(4‐pyridone)_4_(H_2_O)_2_][TM^III^(CN)_6_]}, where TM–transition metal, for optical thermometry sensing. ^[^ ^37^ ^]^

Although the reasons for the presence of additional emission bands at different excitation wavelengths are often sophisticated, ^[^ ^38^, ^39^, ^40^ ^]^ we attempted to identify them relying on the accumulated knowledge of the constitution [Ln(MeDPQ)_2_Cl_3_].

Since Ho^3+^ and Er^3+^ ions have five excited states that are energetically not far apart, so‐called ladder‐type, ^[^ ^37^ ^]^ systems with these ions have more options for energy transfer and nonradiative transitions. Energy gap between the lowest excited state, ^5^F_5_ for the Ho^3+^ ion (∼15 480 cm^−^^1^) and ^4^F_9/2_ for the Er^3+^ (∼15 240 cm^−^^1^), ^[^ ^36^ ^]^ and the underlying level, ^5^I_4_ for the Ho^3+^ ion (∼13 280 cm^−^^1^) and ^4^F_9/2_ for the Er^3+^ ion (∼12 430 cm^−^^1^), ^[^ ^36^ ^]^ is small, making intersystem conversion and energy transfer preferable. Therefore, if the system emits photons with the energy corresponding to the energy gap between the Ln^3+^ states, this results in an increased probability of photon reabsorption by the Ho^3+^ or Er^3+^ ion.

Beyond that, expanded π–systems (phenanthrene, pyrene, perylene, and related compounds) can lead to the formation of exciplexes and excimers in solid state and solution. ^[^ ^40^, ^41^ ^]^ In the case of a suitable position, which enables aromatic moieties to be parallelly oriented to each other at a reasonable distance (less than 4 Å) between the planes, exciplexes can form. ^[^ ^13^, ^39^, ^40^, ^41^, ^42^ ^]^ Furthermore, these phenomena sometimes lead to the formation of rigid covalent bonds between C atoms upon UV irradiation, yielding stable molecules. ^[^ ^43^ ^]^

**Figure 10:**
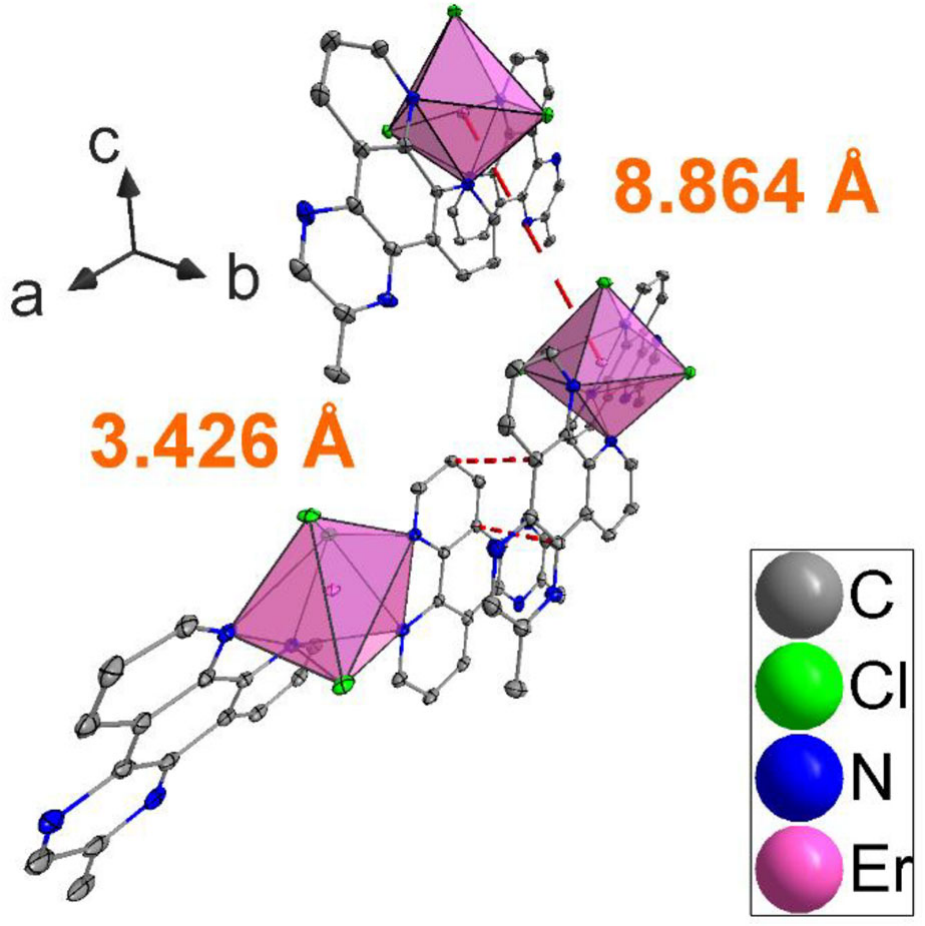
Representation of short contacts between two MeDPQ molecules (3.4258(45) Å) and distance between two Er atoms (8.8636(7) Å) in the crystal structure.

The combination of a larger number of the Ln^3+^ energy levels and favorable conditions for the ligand MeDPQ to stack (Figure 10) can result in the formation of exciplex states and their subsequent radiative deactivation. Our experiments demonstrated that singlet state emission of MeDPQ occurs in the 365–430 nm range (Figure 6), exhibiting transitions S_1_→S_m_ at λ_ex_ = 356 nm and λ_ex_ = 320 nm with the maximum intensity at λ_em_ = 392 nm and λ_em_ = 394 nm in the case of the Ho^3+^ and Er^3+^ complexes, respectively. Since the additional emission bands were observed upon excitation with a longer wavelength (>400 nm), a conventional excitation pathway S_n_←S_0_ cannot be favorable even for n = 1. In addition, these emission bands cannot be exclusively assigned to the triplet state T_1_ of MeDPQ (*E* ≈ 22 600 cm^−^^1^ at 77 K ^[^ ^18^ ^]^) for two reasons. First, at wavelengths > 400 nm absorbance of the ligand has almost the same magnitude as the Ln^3+^‐based one (Figures 8, S20, S21, S24, S25). This indicates that the ligand‐based and the Ln^3+^‐based absorption are competitive processes at these wavelengths. Second, the excitation amplitude of the observed transitions leading to the additional emission diminished tenfold for [Ho(MeDPQ)_2_Cl_3_] (at room temperature and 77 K, Figures 9, S9–S10) and for [Er(MeDPQ)_2_Cl_3_] (only at 77 K) at λ_ex_ < 355 nm (Figure S13). However, the excitation spectrum of [Y(MeDPQ)_2_Cl_3_] (Figure 6) and the excitation spectrum of [Gd(MeDPQ)_2_Cl_3_] indicate that the energy is delivered to the ligand excited states at λ_ex_ < 355 nm. In our previous study of the complexes [Y(MeDPQ)_2_Cl_3_] and [Gd(MeDPQ)_2_Cl_3_], we have proved that this emission originates from the triplet state of the ligand MeDPQ, as the emission lifetimes at room temperature were determined to be 7.8 ms and 0.6 ms, respectively. ^[^ ^2^ ^]^ In the case of [Ho(MeDPQ)_2_Cl_3_], the emission lifetime of λ_em_ = 525 nm was below the instrumental limit with a microsecond flash lamp.

For the comparable complex [HoCl_3_(ptpy)(py)], ptpy − 4′‐phenyl‐2,2′:6′,2′′‐terpyridine, exhibiting exciplex emission, the emission lifetime was determined to have a value of 3.00(1) ns at 77 K. ^[^ ^13^ ^]^ Moreover, exciplex‐based emission of the related ligand system was observed in the dimethylformamide solution (1:500) of the complex *cis*‐[Ir(phen)_2_Cl_2_]^+^ (phen = 1,10‐phenanthroline) and naphthalene. ^[^ ^41^ ^]^

In the case of other literature examples ^3^_∞_[Ln(4‐PyPz)_3_] and ^3^_∞_[Ln(3‐PyPz)_3_] (Ln – Ho, Er; PyPz – pyridilpyrasole) ^[^ ^44^ ^]^ and ^3^_∞_[Ln(3‐PyPzH)_3_Cl_6_]·MeCN (Ln = Ho, Er), ^[^ ^4^ ^]^ neither additional ligand‐based emission, nor *f–f* re‐absorption bands affecting the emission profile of these compounds were reported. Although these compounds demonstrated characteristic *f–f* absorption of Ho^3+^ and Er^3+^, they did not exhibit reabsorption in the visible range and additional ligand‐based emission. In addition, related reabsorption effects were observed in the inorganic structures, such as Ln‐substituted antimonotungstates (AMTs)  . ^[^ ^45^ ^]^

Therefore, we conclude that the suitable crystal packing, a larger number of energy levels of the Ho^3+^ and Er^3+^ ions, and the re‐absorption effects lead to the formation of the exciplex states whose emission was observed.

**Figure 11:**
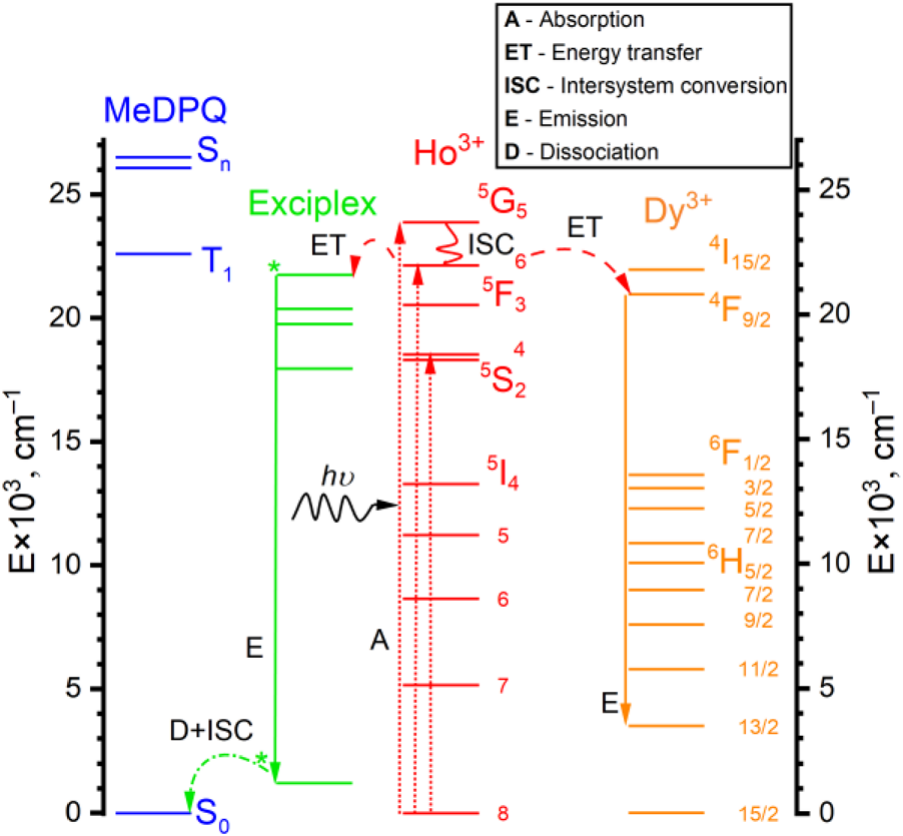
Jablonski diagram of the possible transitions in the substituted complex [Ho_0.5_Dy_0.5_(MeDPQ)_2_Cl_3_]. Internal conversion and back energy transfer processes are omitted for clarity. An asterisk (*) corresponds to the energy levels of the excited complex, which could not be defined. The values of energy levels corresponding to Ln^3+^ states are taken from the fundamental work by W. Carnall and coworkers. ^[^ ^36^ ^]^

A diagram in Figure 11 represents possible pathways leading to the exciplex emission in the substituted complex [Ho_0.5_Dy_0.5_(MeDPQ)_2_Cl_3_]. Initially, switching to a longer excitation wavelength, λ_ex_ = 405 nm, leads to competitive Ho^3+^‐based absorption besides the ligand‐based one (Figures 8, S24). Once the radiation is gathered by the Ho^3+^ ion and its excited states ^5^G_5‐6_ become more populated, two favorable pathways can proceed.

First, absorbed energy can lead to overlap between orbitals of the ligand molecules and subsequent association of the moieties, resulting in additional excited states. ^[^ ^40^ ^]^ Afterward, radiative deactivation of these states can happen, with subsequent dissociation and conversion to the ground state. Moreover, the reabsorption of emitted photons is driven by Ho^3+^ ions, as these transitions were detected (Figures 9, S9, S10). Alternatively, energy transfer proceeds to the ^4^F_9/2_ state of the Dy^3+^ ion with subsequent relaxation to the state ^6^H_15/2_, which results in the weak yet noticeable emission (Figures 9, S17).

### 2.4 Thermal Stability

The behavior of the complexes [Ho(MeDPQ)_2_Cl_3_], [Er(MeDPQ)_2_Cl_3_], and [Er_0.5_Dy_0.5_(MeDPQ)_2_Cl_3_] at elevated temperatures was investigated by simultaneous thermal analysis. Figures S26, S27 show that the compounds [Ho(MeDPQ)_2_Cl_3_] and [Er(MeDPQ)_2_Cl_3_] are stable up to 365°C in the synthetic air atmosphere and then start oxidizing with large heat release. The following thermal effects can correspond to afterburning of carbon residues or phase transition between β‐Ln_2_O_3_ (monoclinic, space group *C*2*/m*) and α‐Ln_2_O_3_ (cubic, space group *Ia*3). In the case of [Er(MeDPQ)_2_Cl_3_], the experimental residual mass of 25.37% (Figure S27) and the theoretical value of 24.96% (Er_2_O_3_, *M* = 382.52 g × mol^−^^1^) correlate well with each other.

To confirm that isomorphous substitution does not affect the thermal stability of the materials, we investigated their properties using the substituted complex [Er_0.5_Dy_0.5_(MeDPQ)_2_Cl_3_] as an example. Figure S28 demonstrates that the substituted compound is as stable as the individual complexes, starting to oxidize at 410°C with a large exo‐effect.

## 3 Conclusion

We have expanded the knowledge about the isotypic complexes [Ln(MeDPQ)_2_Cl_3_] to the lanthanide ions Dy^3+^, Ho^3+^, and Er^3+^ as well as to the statistically substituted complexes [Ln_1‐_ *_x_* Dy*_x_* (MeDPQ)_2_Cl_3_] based on them. Magnetic investigations showed unsaturated stated of the Dy^3+^ ion due to single ion anisotropy and weak magnetic interactions in the crystal structure. The complexes [Ln(MeDPQ)_2_Cl_3_] (Ln = Ho^3+^; Er^3+^) showed complex excitation and emission profiles due to the reabsorption effects. Substitution of Dy^3+^ ions with Ho^3+^ and Er^3+^ ions resulted in the solid solutions Ho_0.5_Dy_0.5_ and Er_0.5_Dy_0.5_, which demonstrated light absorption in the UV, visible, and NIR ranges. Upon excitation with the radiation λ_ex_ > 400 nm, the compounds exhibited additional ligand‐based luminescence and Dy^3+^ emission, indicating the possible energy transfer Ho^3+^/Er^3+^→Dy^3+^. We assigned the nature of the additional emission to the exciplex states that can be formed by the association of the ligand molecules, suitably oriented in the unit cell. Thus, we have experimentally validated that intermolecular coupling between Ln^3+^ ions can occur in mononuclear complexes in the solid state through the aromatic system of the ligands or directly; that is, the distance of 8.9 Å between the ions in the crystal structure is sufficient.

With this work, we have demonstrated that isomorphous substitution in the constitution [Ln(MeDPQ)_2_Cl_3_] resulted in the compounds, which can be utilized as multifunctional materials, possessing visible and NIR emission, absorption in the UV, visible, and NIR ranges, temperature‐dependent luminescent properties, magnetic properties, and sufficient thermal stability.

## 4 Experimental Data

### 4.1 Analytical Methods

#### 4.1.1 Single Crystal X‐ray Diffraction

A colorless, plate‐shaped crystal of [Er(MeDPQ)_2_Cl_3_] was mounted on a goniometer using a perfluorinated ether. Data collection from a shock‐cooled single crystal at 100(2) K was performed on a Bruker D8K Venture equipped with a Bruker CMOS–PHOTON III–C14 detector. The diffractometer used Mo‐K_α_ radiation (λ = 0.71073 Å). All data were integrated with SAINT, and a multi‐scan absorption correction using SADABS was applied. ^[^ ^46^ ^]^ The structure was solved by direct methods with shelXT ^[^ ^47^ ^]^ and refined by full‐matrix least‐squares methods against *F^2^* using shelXL ^[^ ^48^ ^]^ on the graphical platform shelXle. ^[^ ^49^ ^]^ All nonhydrogen atoms were refined with anisotropic displacement parameters. All hydrogen atoms were refined freely with their U_iso_ values constrained to 1.5 times the U_eq_ of their pivot atoms for terminal *sp* ^3^ carbon atoms and 1.2 times for all other carbon atoms. FinalCif software was used for the generation and editing process of the report. ^[^ ^50^ ^]^

#### 4.1.2 Powder X‐Ray Diffraction

X‐ray powder diffraction data of the individual phases [Ln(MeDPQ)_2_Cl_3_] and the isotypic substitution complexes [Ln_1‐*x*_Dy_*x*_(MeDPQ)_2_Cl_3_] were collected at room temperature with a Tongda TD 3700 powder diffractometer (Bragg‐Brentano geometry, CuK_α_ radiation λ = 1.54186 Å, NiK_β_ filter) and linear Mythen detector. The step size of 2θ was selected as 0.019°, and the counting time was 2 s per step.

For the Rietveld refinement, all reflections in the diffractograms obtained were indexed with the orthorhombic cell (*Fdd*2) with parameters close to [Yb(MeDPQ)_2_Cl_3_], ^[^ ^18^ ^]^ which was taken as a starting model for Rietveld refinement using TOPAS 4.2 software. ^[^ ^51^ ^]^ The refinements were stable and gave low R‐factors (Tables 1 and 2).

#### 4.1.3 Investigation of Magnetic Properties

The magnetization of the synthesized compounds [Dy(MeDPQ)_2_Cl_3_] (29.8 mg), [Dy_0.9_Y_0.1_(MeDPQ)_2_Cl_3_] (18.4 mg), and [Dy_0.5_Y_0.5_(MeDPQ)_2_Cl_3_] (22.3 mg) was carried out in the Physical Property Measurement System (PPMS‐9) (Quantum Design, USA) equipped with the vibrating sample magnetometry (VSM) option in the temperature range from 5 to 300 K. The magnetic hysteresis loop was measured in a magnetic field up to 9 T. Magnetization curves in zero‐field‐cooled (ZFC) and field‐cooled (FC) regimes at 100 Oe and 1000 Oe were measured in the temperature range of 5–300 K.

#### 4.1.4 Photoluminescence Investigations

The measurements of excitation and emission spectra, as well as overall emission intensity decay times, were completed at room temperature (23 ± 2°C; samples were not additionally thermostatted). To this end, powdered solid samples were mounted on a special Solid Sample Holder (Horiba) between a PTFE foil and a quartz watch glass in air. Afterward, the holder was placed at a 45° angle to the excitation source. Excitation and emission spectra were recorded using the FluoroEssence software on a Fluorolog FL‐221 spectrofluorimeter (HORIBA Jobin Yvon, Kyoto, Japan) equipped with a Xenon Short Arc Lamp (Ushio FL‐450XOFR), double grated monochromator, and photomultiplier tube R928P for the visible range. Excitation and emission spectra were not additionally corrected. Overall emission decay times were measured on the same setup using the FluoroEssence software. A microsecond flashlamp was used for the excitation. The overall emission intensity decay was fitted with the mono‐ or poly‐exponential decay function $I\;\left(t\right)=A+\sum_{i=1}^{n}B_{i}\cdot e^{\left(-t/\tau_{i}\right)}$ using the Origin PRO 2021. ^[^ ^52^ ^]^

The measurements of NIR emission and investigations at 77 K (using a special liquid nitrogen‐filled assembly FL‐1013 by HORIBA) were performed with a second device, the Fluorolog 3 spectrometer (HORIBA), equipped with a dual lamp house, Xe short‐arc lamp (USHIO, 450 W), Xe short‐arc flashlamp (Excelitas FX‐1102, average power 10 W), double‐grated monochromators, two photomultiplier detectors (R928P for the visible range, R5509‐73 cooled down to −80 °C for NIR range), and a TCSPC upgrade. Before the measurements, powder solid samples were filled in synthetic quartz glass tubes in air. Spectra were corrected for the spectral response of monochromators and detectors using correction files provided by the manufacturer. Excitation spectra were additionally corrected for the lamp's spectral distribution using the reference photodiode detector. A long‐pass filters (Newport, cut‐off 830 nm) were used to avoid second‐order light reflection by monochromators.

#### 4.1.5 Simultaneous Thermal Analysis

Simultaneous thermogravimetry‐differential scanning calorimetry analysis was performed in the dynamic flow of argon (20 ml/min) and synthetic air (30 ml/min) mixture using a NETZSCH STA‐409‐PC instrument from 25°C to 1000°C with a heating rate of 5 K/min. To determine the enthalpy of the processes, the equipment was calibrated using standard substances such as In, Sn, Bi, Zn, Al, Ag, Au, and Ni. The integrals of thermal effect peaks were determined using the package Proteus 6 2012.

#### 4.1.6 Vibrational Spectroscopy

IR spectra of compounds in the solid state, preliminary ground in an agate mortar, were recorded on the Agilent Cary 630 FTIR spectrometer on an ATR attachment. Spectral range of measurement: 4000–400 cm^−1^. Number of sample scans: 80. Detector: deuterated triglycine sulfate (DTGS).

#### 4.1.7 Diffuse Reflectance Spectroscopy

Diffuse reflectance spectra were measured on a UV‐2600 spectrophotometer manufactured by Shimadzu (Japan) equipped with an ISR‐2600Plus attachment with an integrating sphere. The instrument has a double‐beam optical scheme and is equipped with halogen (visible and near‐infrared regions) and deuterium (ultraviolet region) light sources. Two types of detectors are used for registration: a photoelectric multiplier FEU of R‐928 type (UV and visible region) and InGaAs detector (near IR region). The range of measured wavelengths is from 220 nm to 1400 nm. Imaging was carried out by the standard method, and BaSO_4_ (99.8%) was used as a reference. According to the diffuse reflectance spectroscopy data, the value of the forbidden zone width was calculated. For the calculation, we used the theory developed by P. Kubelka and F. Z. Munk‐Aussing. ^[^ ^53^ ^]^ According to this theory, the diffuse reflection of the sample depends only on the ratio of the absorption coefficient *K* and the scattering coefficient *S*: *F*(*R*_∞_) = (1‐*R*_∞_)^2^/2*R*_∞_ = *K*/*S*, where F(R_∞_) is the Kubelka‐Munk function; *R*_∞_ is the diffuse reflection from an infinitely thin sample equal to R_sample_/R_BaSO4_. Reflectance was recalculated to absorbance according to the *A *= *lg*(100/*R*) relation.

### 4.2 Synthesis and Analytical Data

#### 4.2.1 Starting Materials

The MeDPQ ligand was synthesized via the preliminary synthesis of 1,10‐phenanthroline‐5,6‐dione according to the literature procedure ^[^ ^54^ ^]^ and subsequent condensation with 1,2‐diaminopropane in the presence of equimolar amounts of H_2_O_2_ to accelerate the oxidation of the unconjugated intermediate. Purification of pyridine was performed according to the literature procedure. CHCl_3_ (99%, stabilized with EtOH 0.5%, *Basa №1 Khimreaktivy*, Moscow, Russia) and ErCl_3_∙6H_2_O (99.999%, ABCR, Karlsruhe, Germany) were used without further purification. DyCl_3_∙6H_2_O and YCl_3_∙6H_2_O (99%, CJSC *Zavod redkikh metallov*, Novosibirsk, Russia) were recrystallized from acidic solutions. HoCl_3_∙6H_2_O was isolated by crystallization from a saturated solution. This solution was obtained by the reaction of the Ho_2_O_3_ oxide with 99.999% purity (LLC *TDM 96*, Yekaterinburg, Russia) and concentrated HCl solution (11.5 M). The phase purity of the starting compounds was confirmed by powder X‐ray phase, and the purity of the ligand was checked by high‐performance liquid chromatography (Knauer Smartline HPLC system), and it was found to be > 98%.

#### 4.2.2 Synthesis of [Ln(MeDPQ)_2_Cl_3_]

All complexes were synthesized under similar conditions: 0.135 or 0.196 mmol of LnCl_3_∙6H_2_O and 0.3 or 0.4 mmol of MeDPQ for the 100 mg and 150 mg product synthesis, respectively, were placed together in borosilicate pressure tubes with a screw cap (Chemglass Life Sciences; volume: 15 or 35 ml, maximum pressure: 150 psi). Then, 3.5 ml of Py for 100 mg synthesis or 5 ml for 150 mg synthesis and a magnetic stir bar were added. The reaction mixture was placed in a silicone bath heated to 125 °C and left for 4–5 hours upon stirring. After the reaction, the contents of the initial tube were transferred to centrifugation tubes through a glass funnel. To achieve quantitative transfer, the initial tubes were washed with 1 mL of Py. After centrifugation (3000 rpm, 10 minutes), the solvent was removed via a syringe and a 100 mm cannula. The next step was repeated twice: 5 ml of CHCl_3_ was added with further stirring, the tubes were centrifuged again, and the solvent was removed. The stirring bar was removed with a magnet after the second centrifugation process. Then, all powders were dried in a vacuum at 60 °C overnight.

[Dy(MeDPQ)_2_Cl_3_] – pale gray powder with yellow luminescence. Yield: **90%** (94.7 mg).

[Y(MeDPQ)_2_Cl_3_] – pale pink powder with yellowish‐green luminescence. Yield: **88%** (87.3 mg).

[Ho(MeDPQ)_2_Cl_3_] – pale yellow powder without visible luminescence. Yield: **92%** (140.6 mg).

[Er(MeDPQ)_2_Cl_3_] – pink powder without visible luminescence. Yield: **89%** (134.2 mg).

#### 4.2.3 Synthesis of [Dy_1‐_ *_x_* Ln*_x_* (MeDPQ)_2_Cl_3_] (Ln = Y^3+^, or Ho^3+^, or Er^3+^)

All complexes were synthesized similarly to the individual phases. For samples with 10% of Y^3+^, 50% of Y^3+^, 50% of Ho^3+^, and 50% of Er^3+^ the following procedure was applied: required stoichiometric quantities of metal salts and borosilicate pressure tubes with a screw cap (Chemglass Life Sciences; volume: 15 or 35 ml, maximum pressure: 150 psi). The next steps were the same as for the individual phases. Actual masses of LnCl_3_∙6H_2_O and MeDPQ for each sample are reported in the Supporting Information (general information section).

[Y_0.9_Dy_0.1_(MeDPQ)_2_Cl_3_] – pale solid with yellowish‐green luminescence. Yield: **85%** (133.9 mg).

[Dy_0.5_Y_0.5_(MeDPQ)_2_Cl_3_] – pale solid with yellow luminescence. Yield: **91%** (137.3 mg).

[Dy_0.5_Ho_0.5_(MeDPQ)_2_Cl_3_] – pale solid with weak yellow luminescence. Yield: **90%** (130.5 mg).

[Dy_0.5_Er_0.5_(MeDPQ)_2_Cl_3_] – pale solid with weak yellow luminescence. Yield: **88%** (138.9 mg).

#### 4.2.4 Single‐Crystal Synthesis

To obtain single crystals of [Er(MeDPQ)_2_Cl_3_], 0.007 mmol (2.7 mg) of ErCl_3·_6H_2_O and 0.016 mmol (4.0 mg) of MeDPQ were placed into a Duran glass ampule (outer ø 10 mm, wall thickness 1.5 mm). Afterward, 0.45 ml of pyridine was added via a syringe, and the ampule was sealed under vacuum (0.008 mbar). The ampule with the resulting mixture was placed inside a resistance heating oven with thermal control (Eurotherm 2416), which was heated to 150°C within 8 hours, then the temperature was held for 8 hours, and then cooled down to room temperature in 4 hours. The resulting crystals were suitable for single‐crystal X‐ray diffraction.

Deposition Number(s) 2463916 contain(s) the supplementary crystallographic data for this paper. These data are provided free of charge by the joint Cambridge Crystallographic Data Centre and Fachinformationszentrum Karlsruhe Access Structures service.

## Supporting Information

This information includes general details, vibrational spectra, detailed crystallographic data, magnetization curves, deciphered photoluminescent and diffuse reflectance spectra, and TG/DSC curves (27 pages). CCDC 2463916 contains the supplementary crystallographic data for this paper. These data can be obtained free of charge from the Cambridge Crystallographic Data Centre via www.ccdc.cam.ac.uk/structures.

## Conflict of Interest

There are no conflicts to declare.

## Supporting information



Supporting Information

Supporting Information

## Data Availability

The data supporting this article are available in the supplementary material of this article.
