# Supplementary material for: Effects of Isomorphous Substitution on Photophysical and Magnetic Properties of Complexes [Ln1‐ x Dy x (MeDPQ)2Cl3] (Ln = Y3+, Ho3+, and Er3+)
Source: Chemistry. 2025 Oct 23;31(66):e02070. doi: 10.1002/chem.202502070 (PMC12648457; doi:10.1002/chem.202502070)
Supplement: Supplementary file 1 — Supporting Information [file CHEM-31-e02070-s001.docx]

**Supporting Information**

**Effects of Isomorphous Substitution on Photophysical and Magnetic Properties of Complexes [Ln_1-_*_x_*Dy*_x_*(MeDPQ)_2_Cl_3_] (Ln = Y^3+^, Ho^3+^, and Er^3+^)**

Maksim A. Zhernakov,^1,2*^ Yuriy G. Denisenko,^1,3,4^ Ruslan G. Batulin,^5^ Ildar. I. Mirzayanov,^2,6^ Elza D. Sultanova,^2^ Maxim S. Molokeev,^7,8,9^ Vladimir A. Burilov,^2^ Valery G. Shtyrlin,^2*^ and Klaus Müller-Buschbaum^1,10^

1 – Institute of Inorganic and Analytical Chemistry, Justus-Liebig-University Giessen, Heinrich-Buff-Ring 17, 35392 Giessen, Germany

2 – A. M. Butlerov Chemistry Institute, Kazan Federal University, Kremlevskaya St., 18, Kazan 420008, Russia

3 – School of Natural Sciences, Tyumen State University, Tyumen 625003, Russia

4 – Department of Construction Materials, Industrial University of Tyumen, Tyumen 625000, Russia

5 – Institute of Physics, Kazan Federal University, Kremlevskaya St., 18, Kazan 420008, Russia

6 – Department of Analytical Chemistry, Certification and Quality Management, Institute of Petroleum, Chemistry and Nanotechnologies, Kazan National Research Technological University, Karl Marx St., 68, Kazan 420015, Russia

7 – Laboratory of Crystal Physics, Kirensky Institute of Physics Federal Research Center KSC SB RAS, Krasnoyarsk 660036, Russia

8 – Department of Engineering Physics and Radioelectronic, Siberian Federal University, Krasnoyarsk 660041, Russia

9 – Department of Physics, Far Eastern State Transport University, Serysheva str. 47, Khabarovsk 680021, Russia

10 – Center for Materials Research (LaMa), Justus-Liebig-University Giessen, Heinrich-Buff-Ring 16, 35392 Giessen, Germany

**Table of contents**

[General information S3](#_Toc200644512)

[IR Spectra S4](#_Toc200644513)

[Crystal structure S5](#_Toc200644514)

[Magnetic Properties S6](#_Toc200644515)

[Photoluminescent Spectroscopy S12](#_Toc200644516)

[Diffuse Reflectance Spectroscopy S18](#_Toc200644517)

[Thermal Analysis S26](#_Toc200644518)

General information

All synthetic work was done on air. Schlenk line was used for sealing ampules. Analytical scales (VIBRA AF225DRCE, Shinko Denshi, Japan) with a readability of 0.00001 g (0.01 mg) were used for sample preparations.

The following masses of the compounds were mixed together:

[Dy(MeDPQ)_2_Cl_3_] – 0.138 mmol (52.1 mg) of DyCl_3_∙6H_2_O and 0.309 mmol (76.0 mg) of MeDPQ

[Y(MeDPQ)_2_Cl_3_] – 0.144 mmol (43.7 mg) of YCl_3_∙6H_2_O and 0.300 mmol (74.6 mg) of MeDPQ

[Ho(MeDPQ)_2_Cl_3_] – 0.199 mmol (75.5 mg) of HoCl_3_∙6H_2_O and 0.430 mmol (105.8 mg) of MeDPQ

[Er(MeDPQ)_2_Cl_3_] – 0.196 mmol (74.7 mg) of ErCl_3_∙6H_2_O and 0.400 mmol (100.8 mg) of MeDPQ

[Y_0.9_Dy_0.1_(MeDPQ)_2_Cl_3_] – 0.200 mmol (60.7 mg) of YCl_3_∙6H_2_O, 0.026 mmol (9.9 mg) of DyCl_3_∙6H_2_O, and 0.453 mmol (111.7 mg) of MeDPQ

[Dy_0.5_Y_0.5_(MeDPQ)_2_Cl_3_] – 0.104 mmol (31.4 mg) of YCl_3_∙6H_2_O, 0.109 mmol (41.0 mg) of DyCl_3_∙6H_2_O, and 0.439 mmol (108 mg) of MeDPQ

[Dy_0.5_Ho_0.5_(MeDPQ)_2_Cl_3_] – 0.095 mmol (35.9 mg) of HoCl_3_∙6H_2_O, 0.095 mmol (35.9 mg) of DyCl_3_∙6H_2_O, and 0.413 mmol (101.7 mg) of MeDPQ

[Dy_0.5_Er_0.5_(MeDPQ)_2_Cl_3_] – 0.104 mmol (39.8 mg) of ErCl_3_∙6H_2_O, 0.102 mmol (38.5 mg) of DyCl_3_∙6H_2_O, and 0.400 mmol (100.4 mg) of MeDPQ

IR Spectra


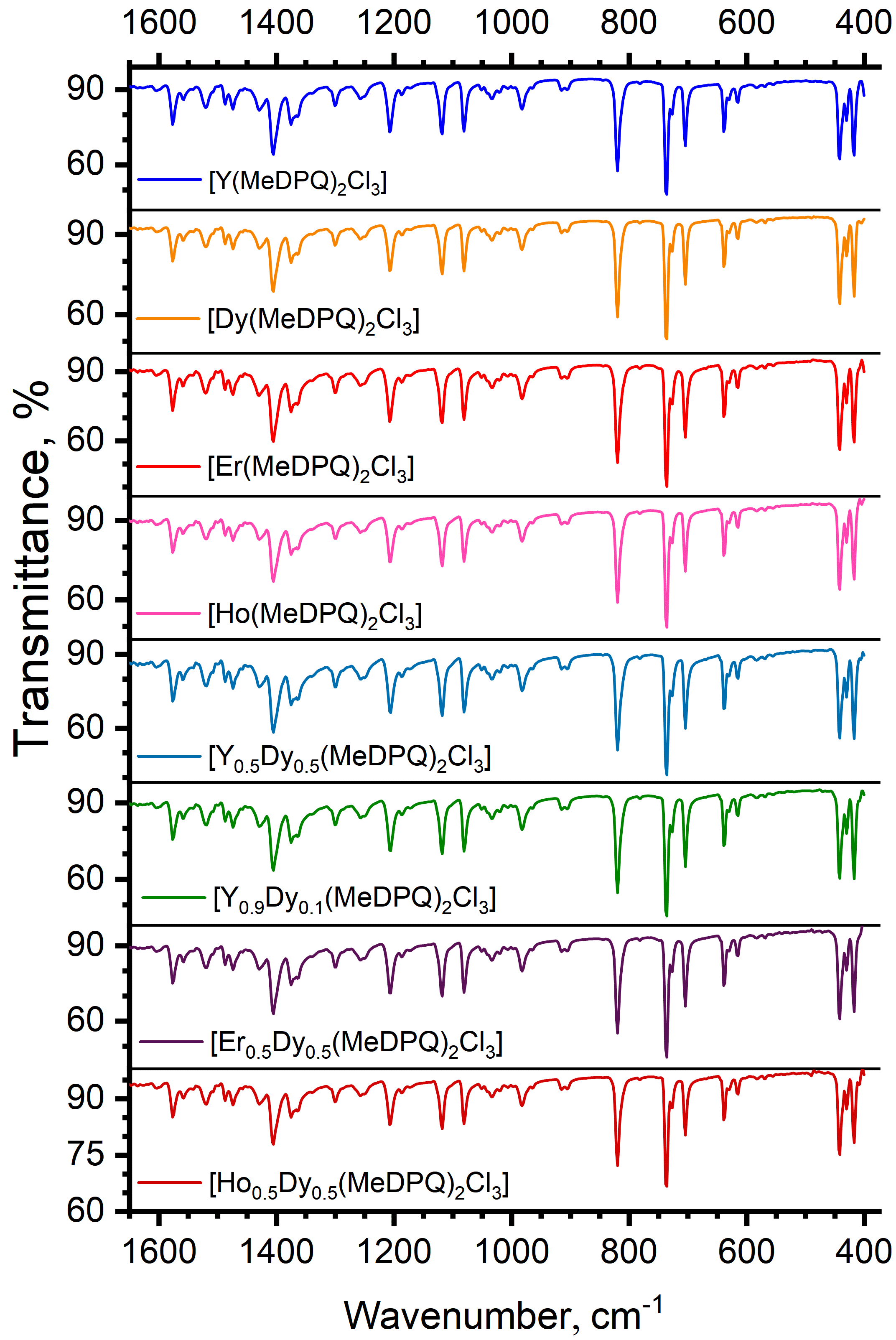


**Figure S1**. IR spectra of the individual complexes [Ln(MeDPQ)_2_Cl_3_] and substitutional complexes
[Ln_1-_*_x_*Dy*_x_*(MeDPQ)_2_Cl_3_].

Crystal structure

**Table S1**. Crystal data and structure refinement parameters of the single crystal [Er(MeDPQ)_2_Cl_3_]

| Empirical formula | C_30_H_20_Cl_3_ErN_8_ |
| --- | --- |
| Formula weight | 766.15 |
| Temperature [K] | 100(2) |
| Crystal system | orthorhombic |
| Space group (number) | *Fdd*2 (# 43) |
| *a* [Å] | 36.790(3) |
| *b* [Å] | 10.1394(9) |
| *c* [Å] | 14.5412(14) |
| α [°] | 90 |
| β [°] | 90 |
| γ [°] | 90 |
| Volume [Å^3^] | 5424.3(8) |
| *Z* | 8 |
| *ρ*_calc_ [g×cm^−3^] | 1.876 |
| *μ* [mm^−1^] | 3.429 |
| *F*(000) | 3000 |
| Crystal size [mm^3^] | 0.088×0.118×0.298 |
| Radiation | Mo*K_α_* (λ=0.71073 Å) |
| 2θ range [°] | 4.43 to 65.35 (0.66 Å) |
| Index ranges | −54 ≤ h ≤ 55 −14 ≤ k ≤ 15 −21 ≤ l ≤ 22 |
| Reflections collected | 37799 |
| Independent reflections | 4952  *R*_int_ = 0.0634 *R*_sigma_ = 0.0373 |
| Completeness to θ = 25.242° | 100.0 % |
| Data / Restraints / Parameters | 4952 / 8 / 221 |
| Absorption correction T_min_/T_max_ (method) | 0.5820 / 0.7464 (multi-scan) |
| Goodness-of-fit on *F*^2^ | 1.074 |
| Final *R* indexes [*I*≥2σ(*I*)] | *R*_1_ = 0.0216 w*R*_2_ = 0.0500 |
| Final *R* indexes [all data] | *R*_1_ = 0.0229 w*R*_2_ = 0.0505 |
| Largest peak/hole [eÅ^−3^] | 0.99/−0.77 |
| Flack X parameter | 0.065(5) |

Magnetic Properties


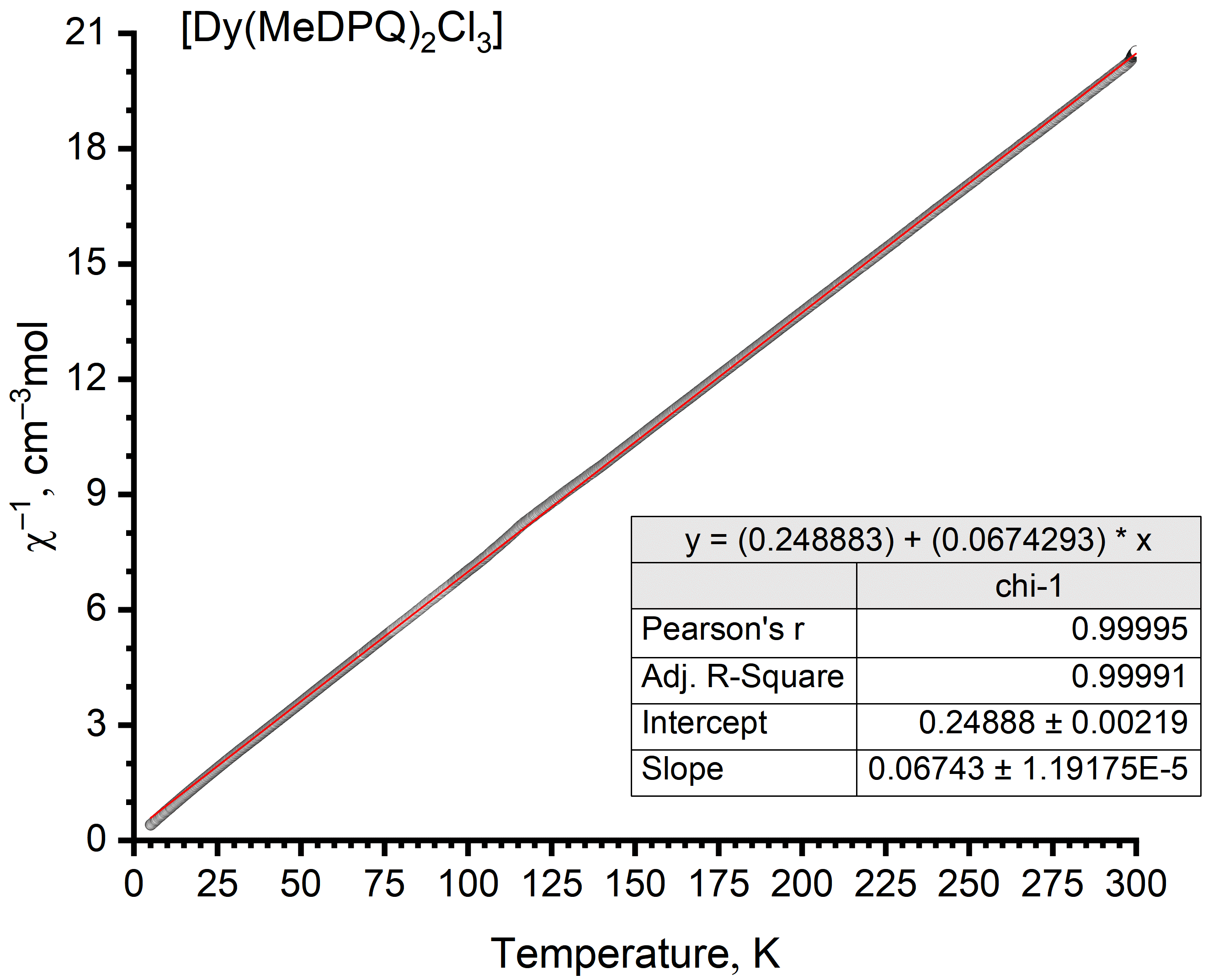


**Figure S2**. Linear approximation (red) of the *χ*^–1^(*T*) data (black) registered in FC mode and at 1000 Oe field for the solid-state compound [Dy(MeDPQ)_2_Cl_3_].


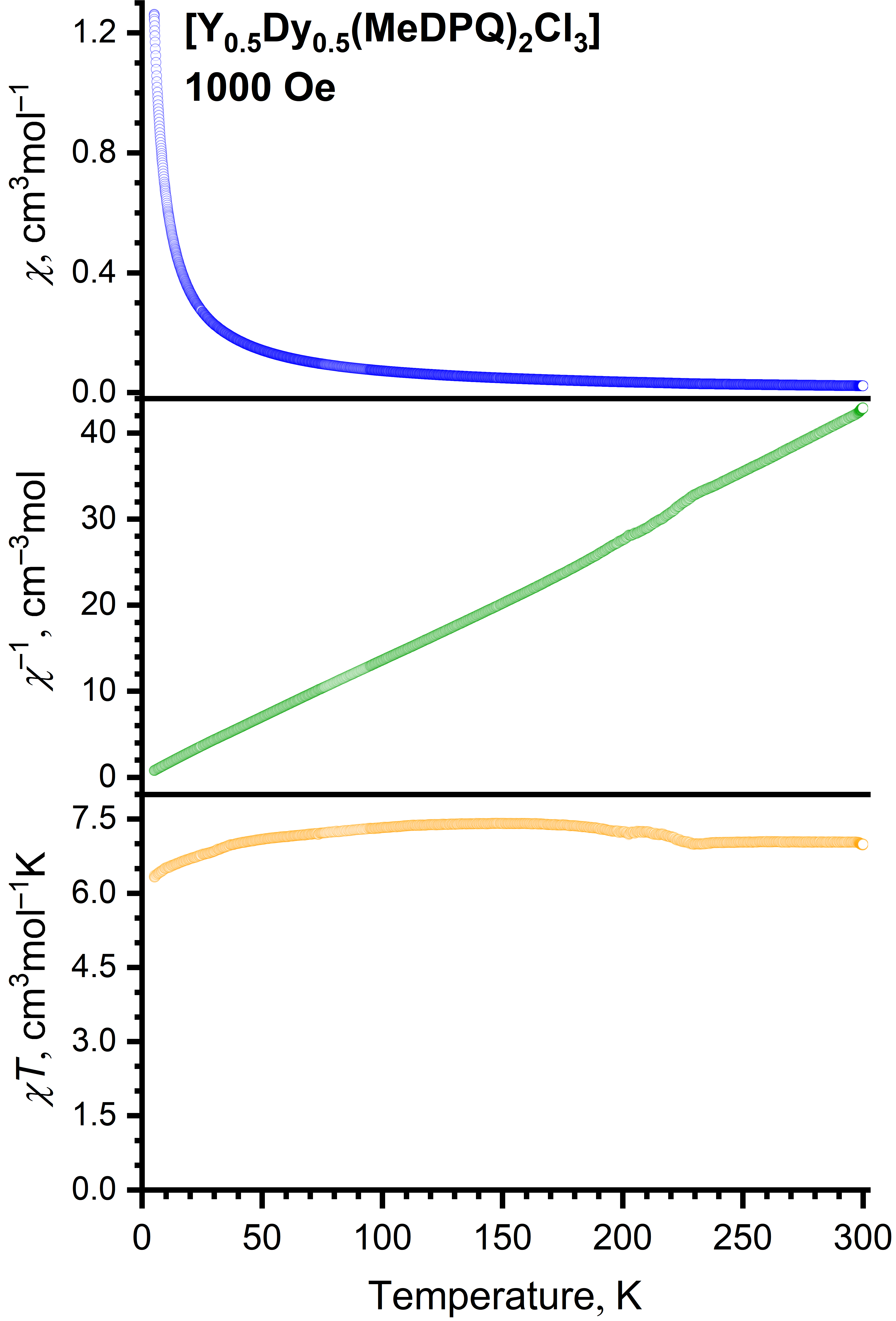


**Figure S3**. Temperature dependence of magnetic susceptibility of the solid-state complex [Y_0.5_Dy_0.5_(MeDPQ)_2_Cl_3_] measured at 1000 Oe and FC mode within the range 5–300 K.


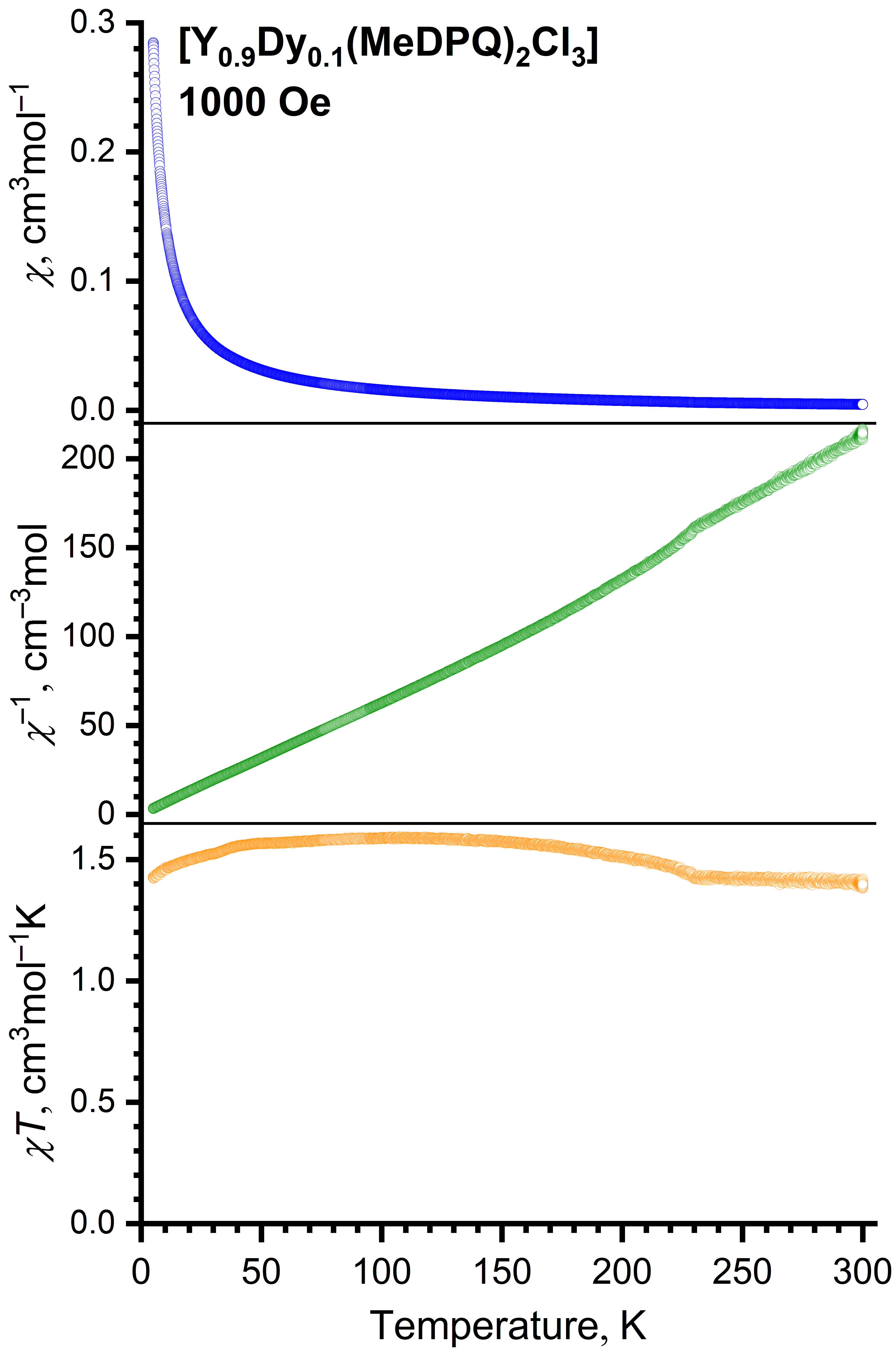


**Figure S4**. Temperature dependence of magnetic susceptibility of the solid-state complex [Y_0.9_Dy_0.1_(MeDPQ)_2_Cl_3_] measured at 1000 Oe and FC mode within the range 5–300 K.


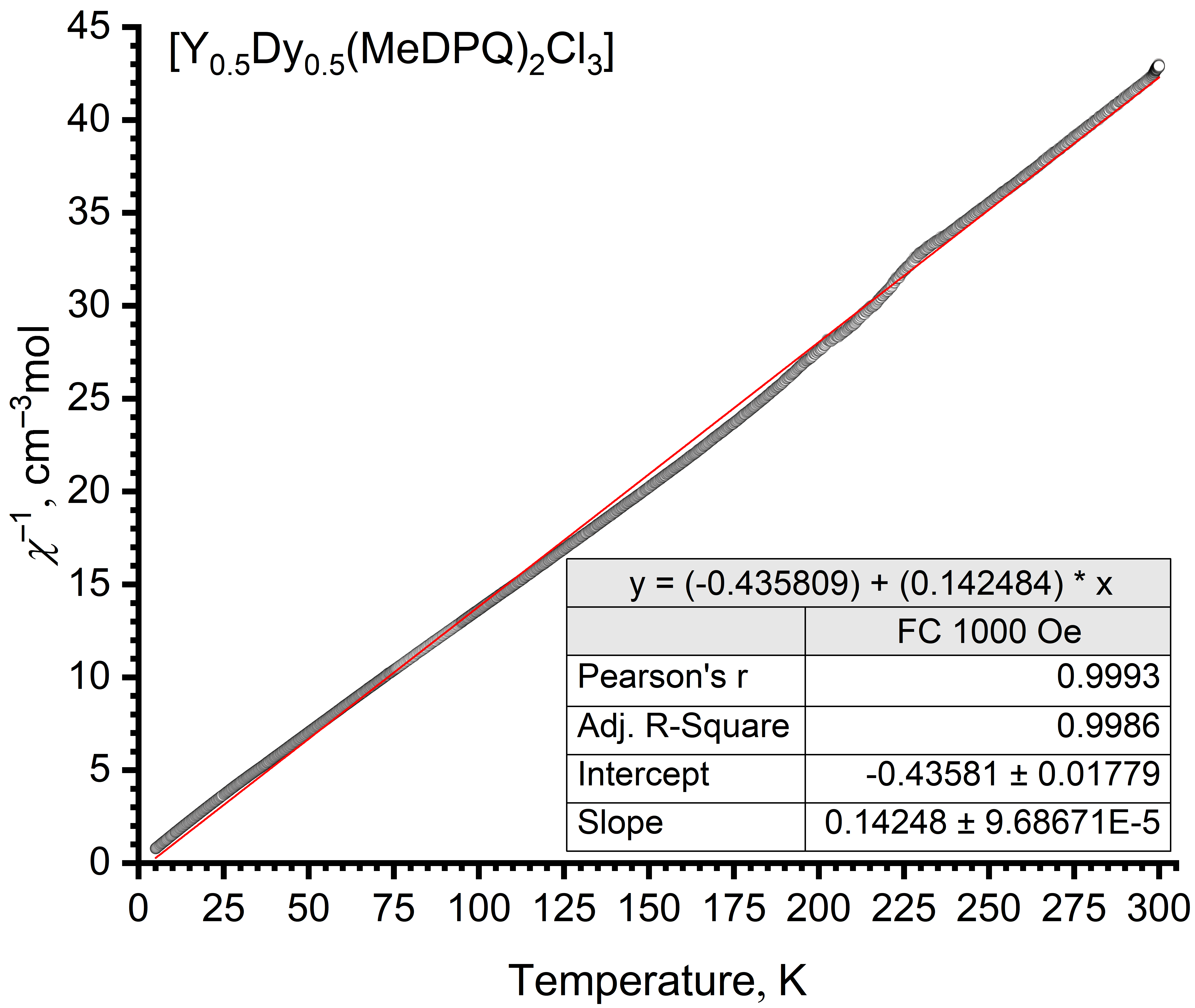


**Figure S5**. Linear approximation (red) of the *χ*^–1^(*T*) data (black) registered in FC mode and at 1000 Oe field for the solid-state compound [Y_0.5_Dy_0.5_(MeDPQ)_2_Cl_3_].


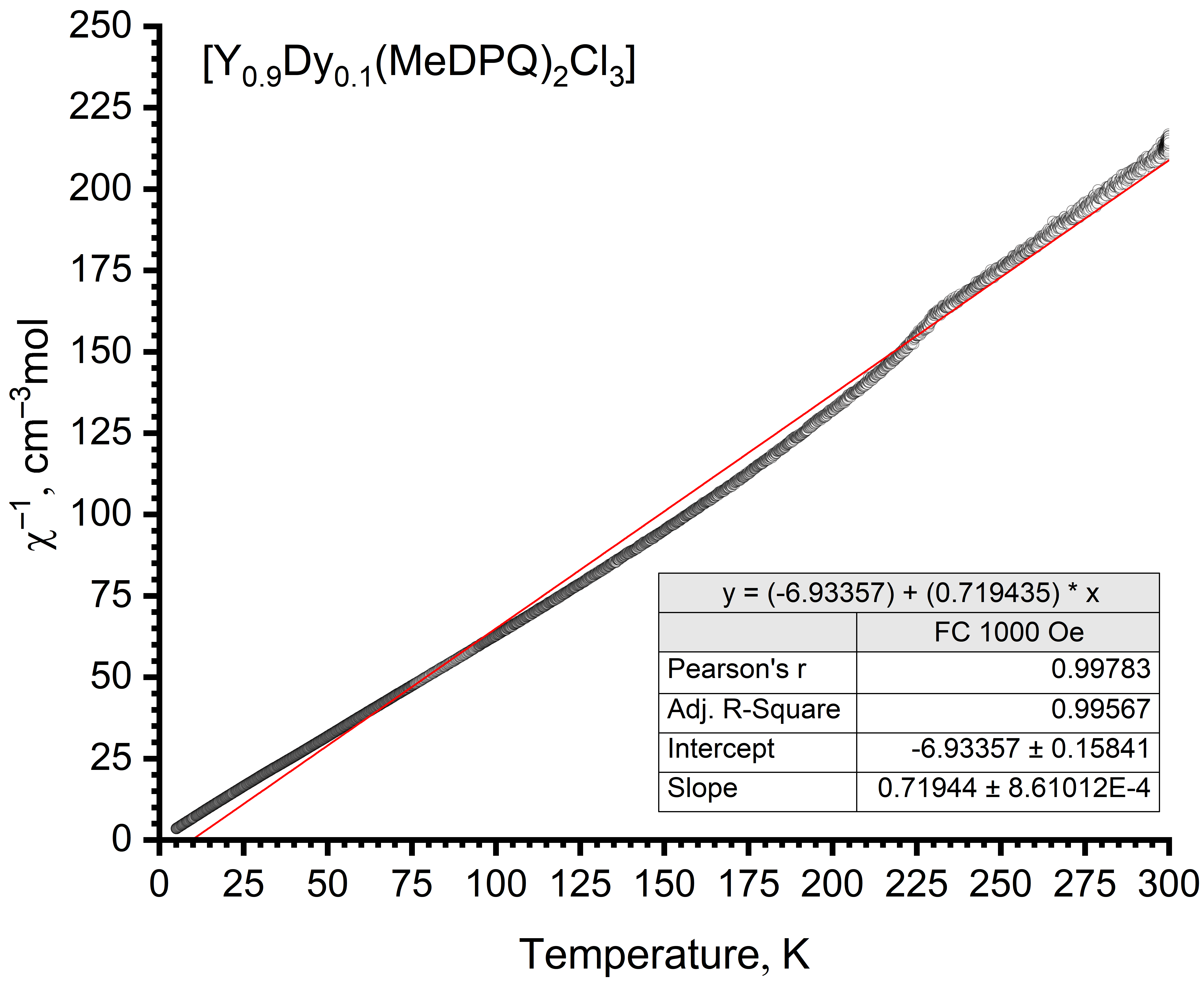


**Figure S6**. Linear approximation (red) of the *χ*^–1^(*T*) data (black) registered in FC mode and at 1000 Oe field for the solid-state compound [Y_0.9_Dy_0.1_(MeDPQ)_2_Cl_3_].

Photoluminescent Spectroscopy


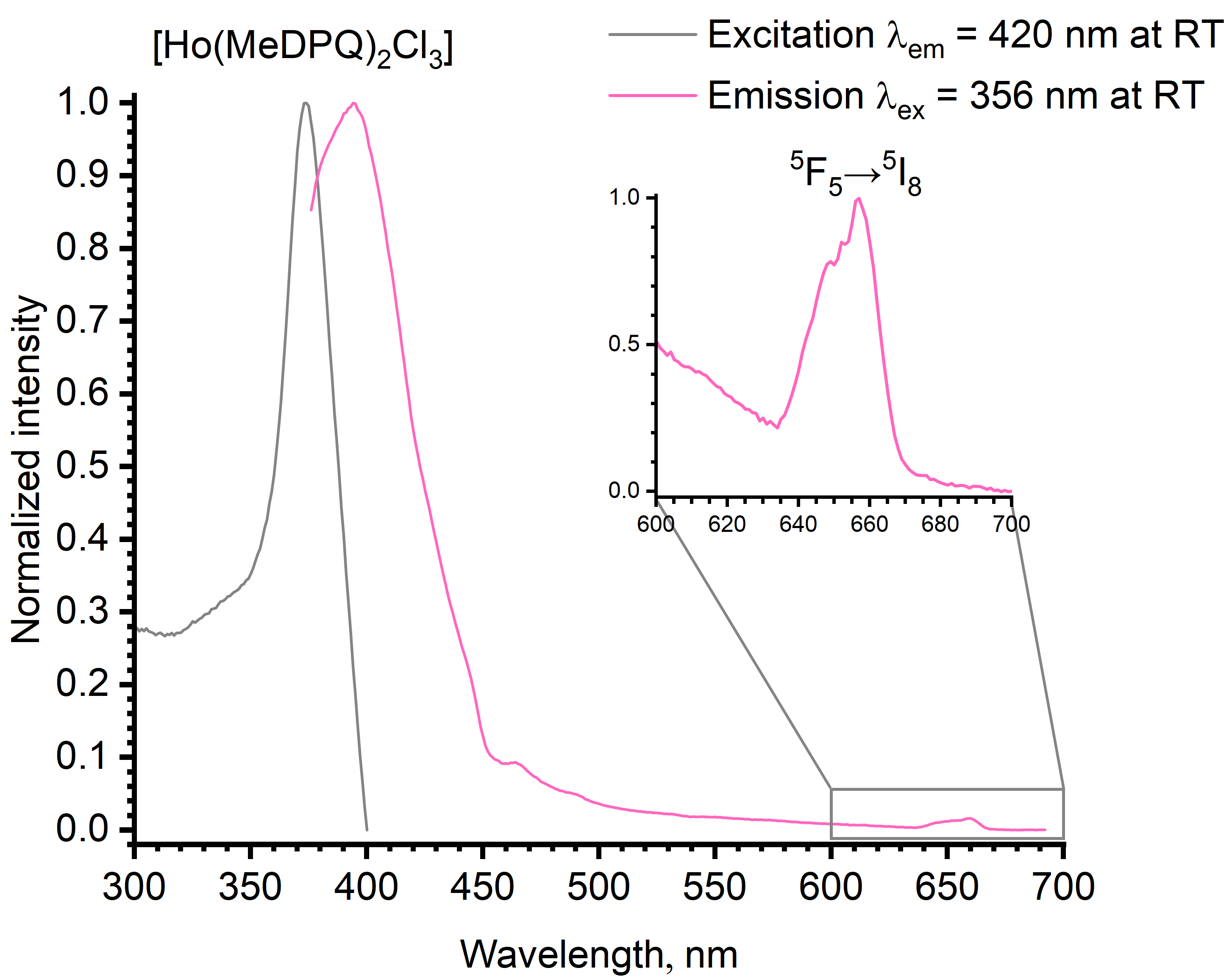


**Figure S7**. Normalized photoluminescence spectra of the solid-state sample [Ho(MeDPQ)_2_Cl_3_] at room temperature. Ho^3+^-based *f*–*f* transition is labelled according to the energy levels.


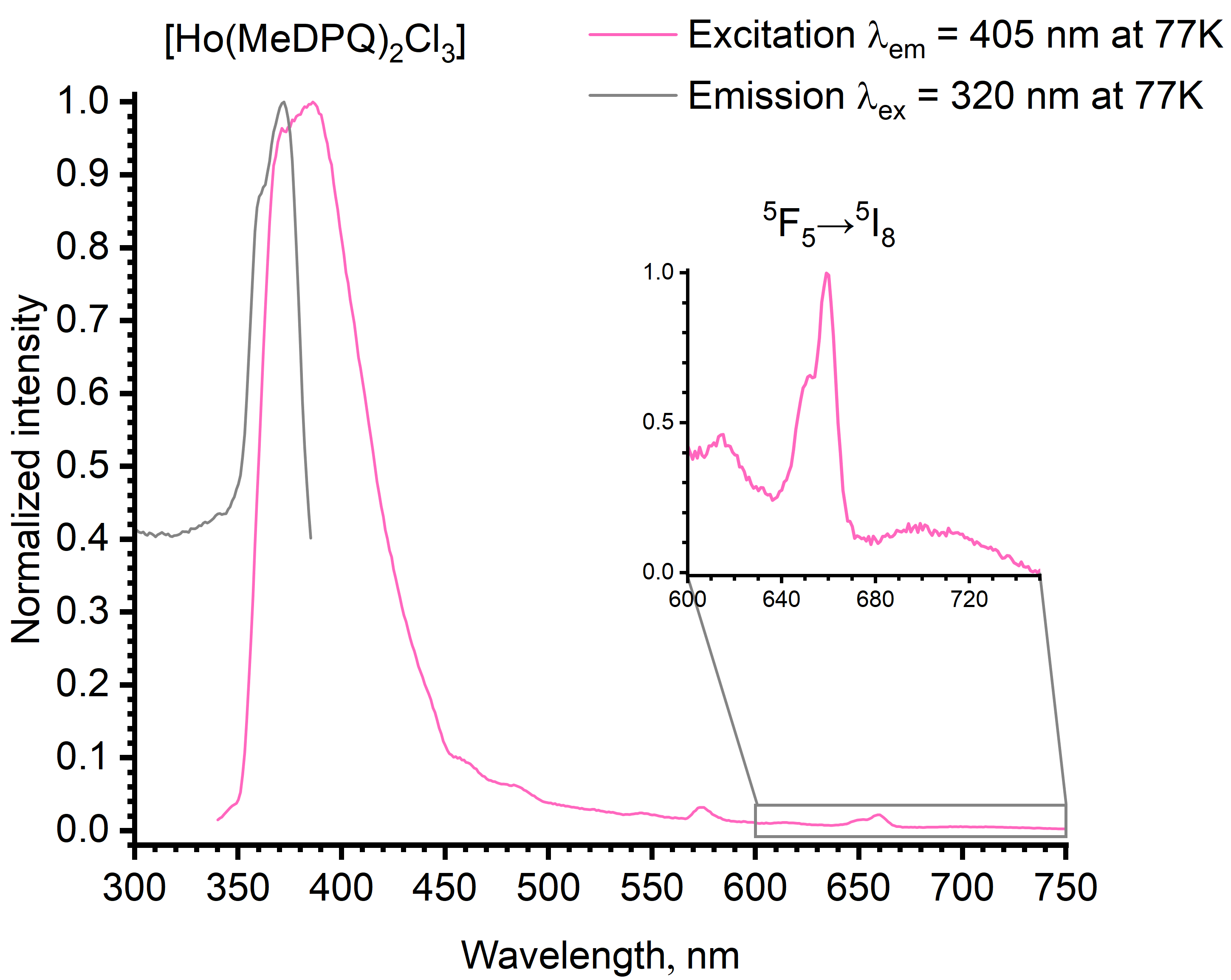


**Figure S8**. Normalized photoluminescence spectra of the solid-state sample [Ho(MeDPQ)_2_Cl_3_] at 77 K. Ho^3+^-based *f*–*f* transition is labelled according to the energy levels. The UV and visible part of the spectra were converged to the same intensity at 574 nm.


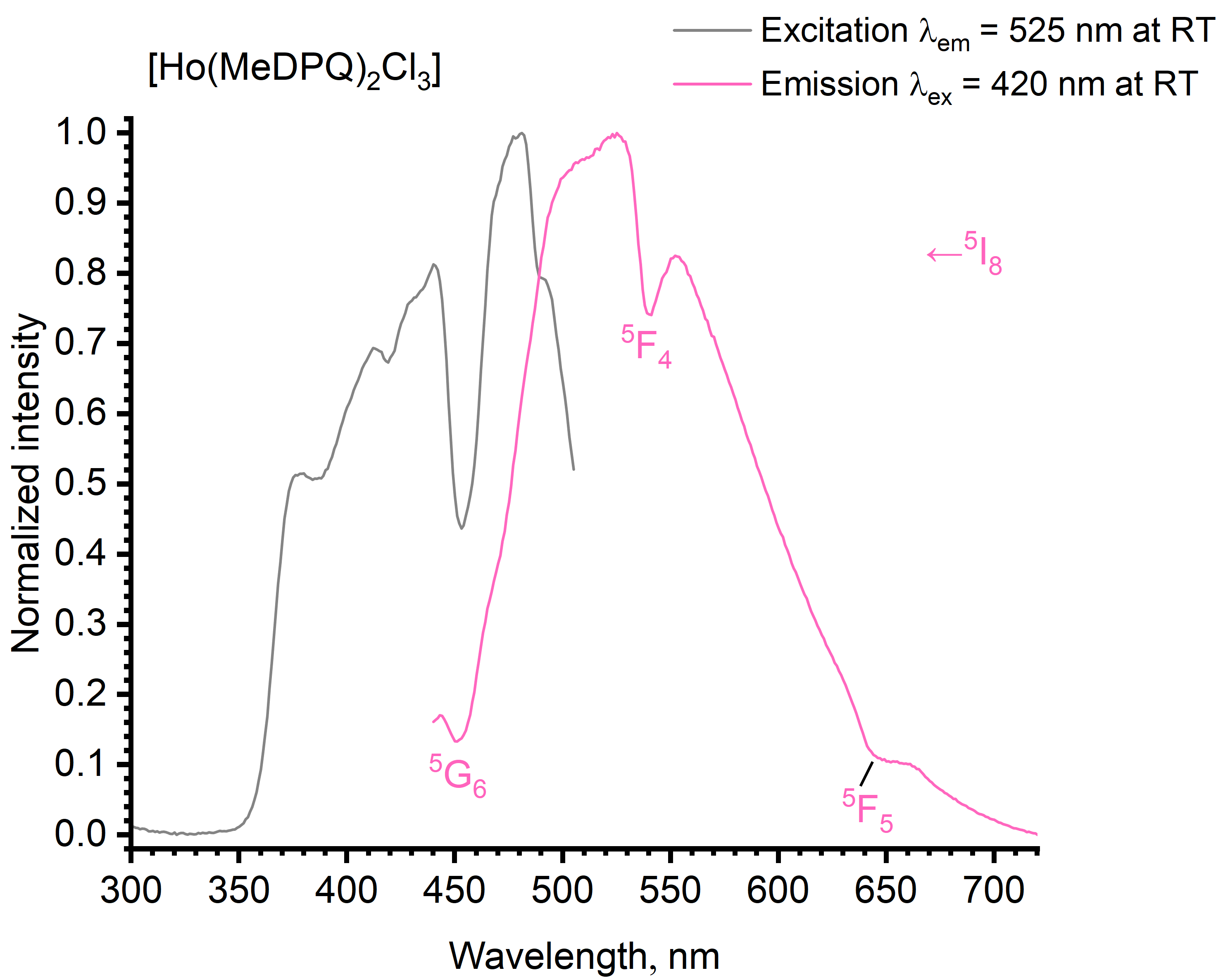


**Figure S9**. Normalized photoluminescence spectra of the solid-state sample [Ho(MeDPQ)_2_Cl_3_] at room temperature, depicting additional ligand-based bands. Ho^3+^-based re-absorption of photons is labelled according to the energy levels.


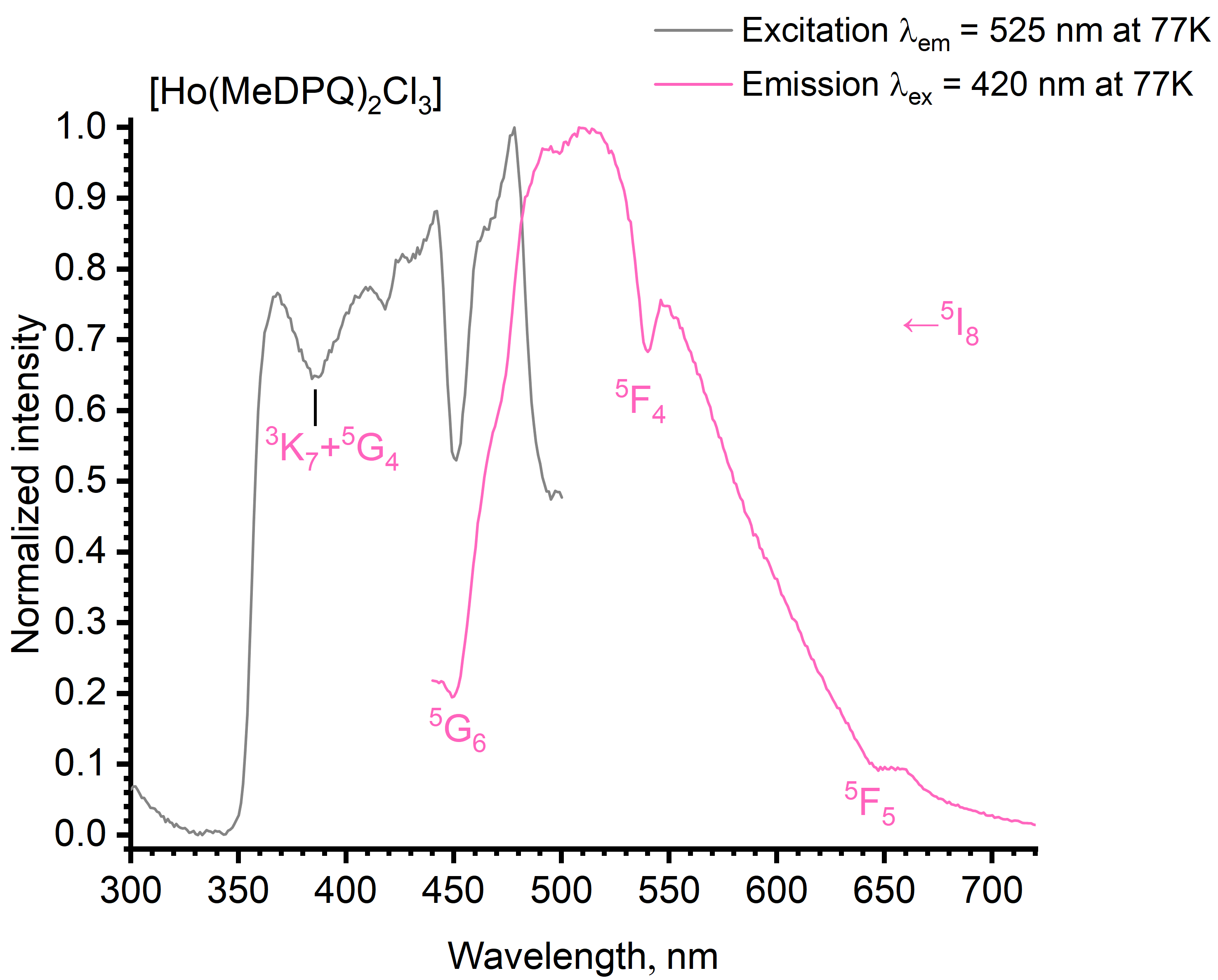


**Figure S10**. Normalized photoluminescence spectra of the solid-state sample [Ho(MeDPQ)_2_Cl_3_] at 77 K, depicting additional ligand-based bands. Ho^3+^-based re-absorption of photons is labelled according to the energy levels.


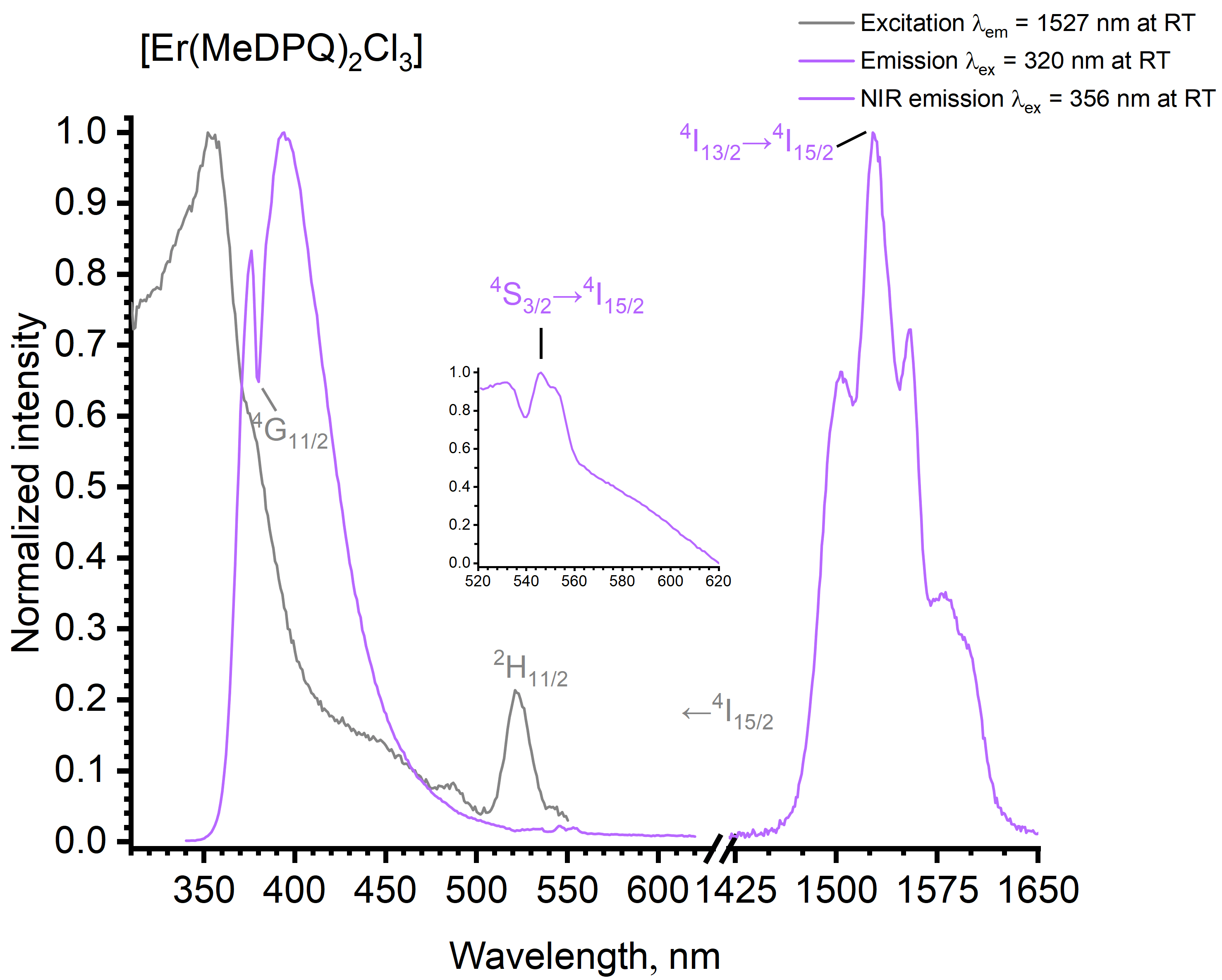


**Figure S11**. Normalized photoluminescence spectra of the solid-state sample [Er(MeDPQ)_2_Cl_3_] at room temperature. The visible and NIR parts of the spectra were normalized separately. Er^3+^-based *f*–*f* transitions are labelled according to the energy levels.


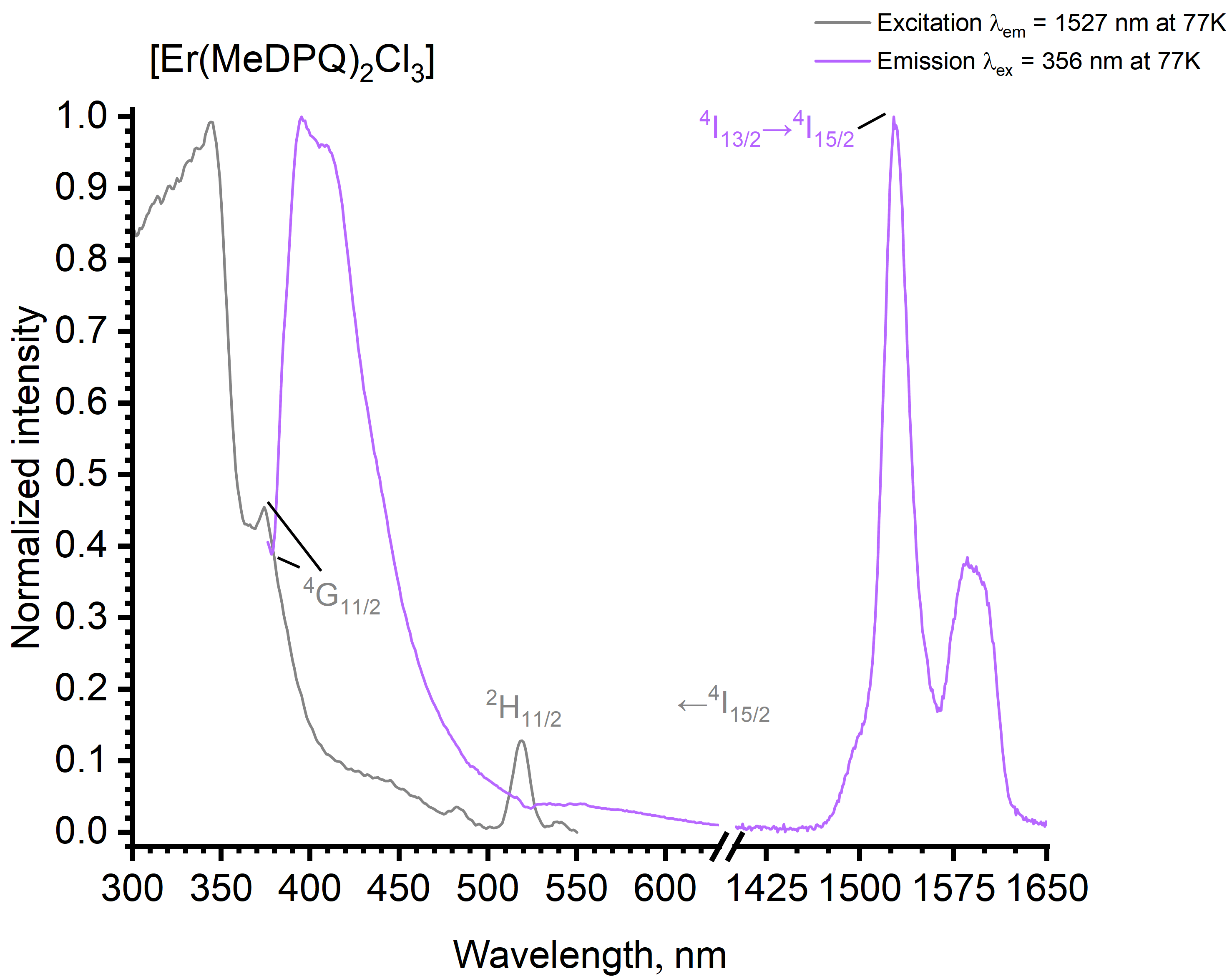


**Figure S12**. Normalized photoluminescence spectra of the solid-state sample [Er(MeDPQ)_2_Cl_3_] at 77 K. The visible and NIR parts of the spectra were normalized separately. Er^3+^-based *f*–*f* transitions are labelled according to the energy levels.


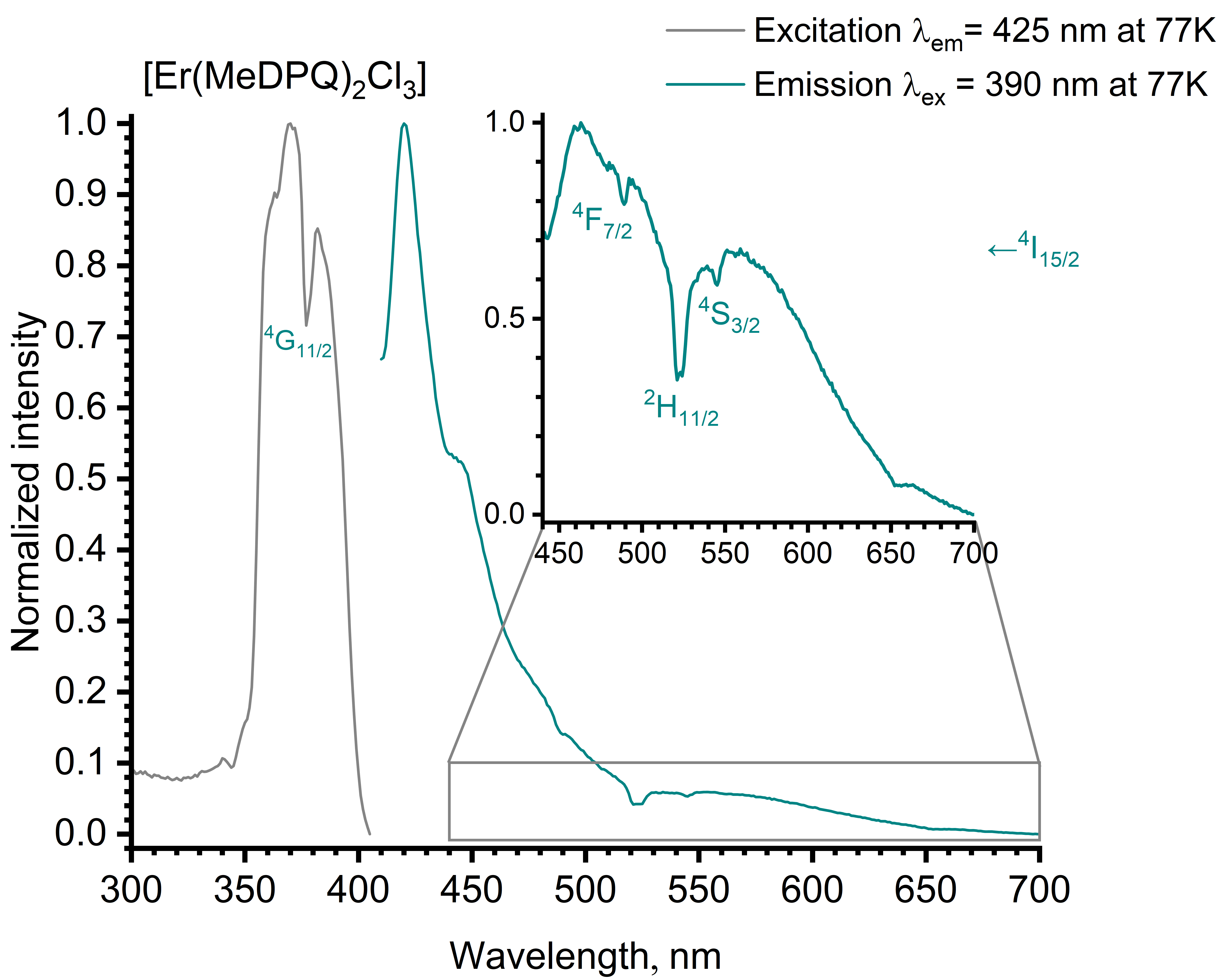


**Figure S13**. Normalized photoluminescence spectra of the solid-state sample [Er(MeDPQ)_2_Cl_3_] at 77 K, demonstrating additional ligand-based bands. The inset spectra were recorded with λ_ex_ = 420 nm. Er^3+^-based re-absorption bands are labelled according to the energy levels.


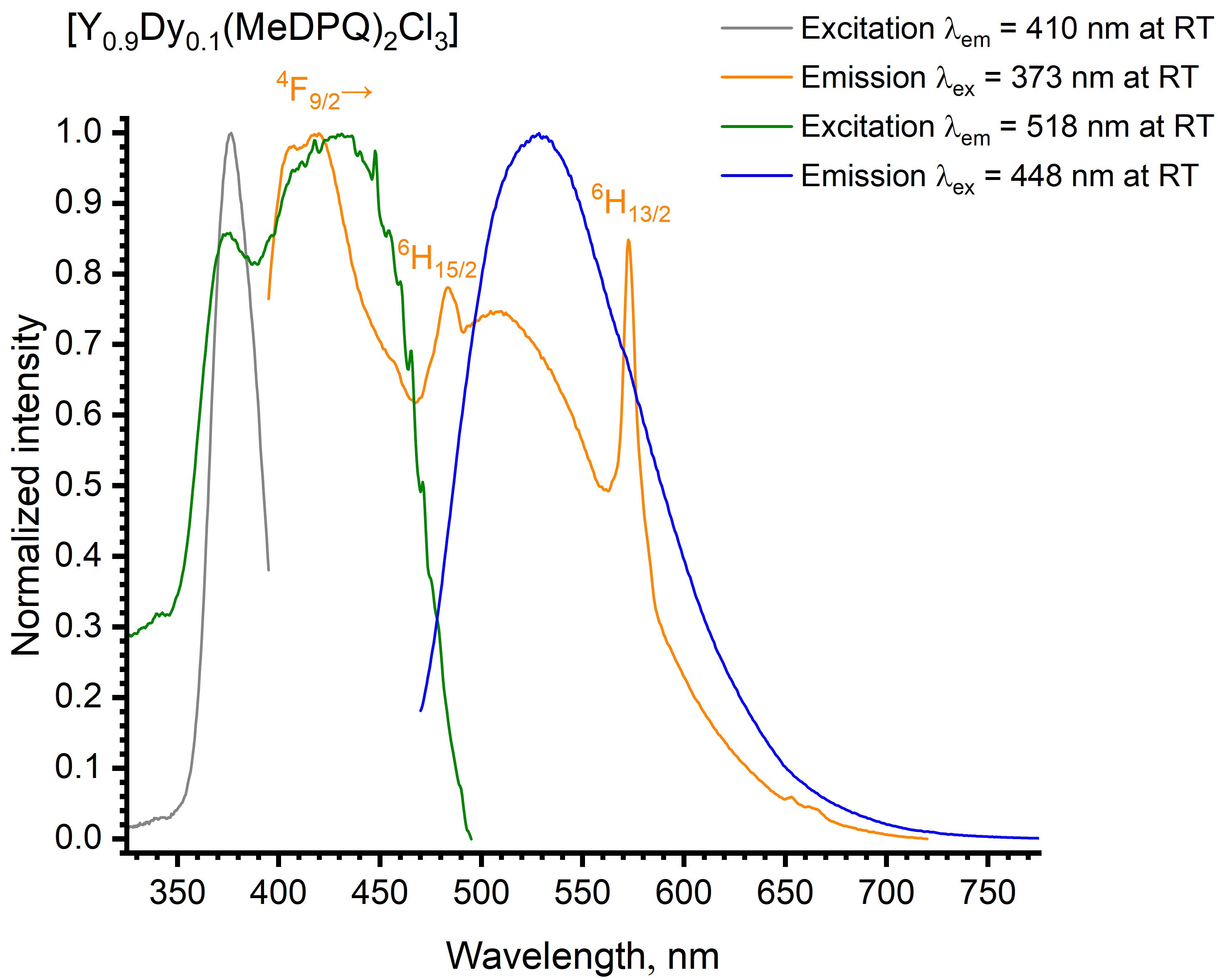


**Figure S14**. Normalized photoluminescence spectra of the solid-state sample [Y_0.9_Dy_0.1_(MeDPQ)_2_Cl_3_] at room temperature. Dy^3+^-based *f*–*f* transitions are labelled according to the energy levels.


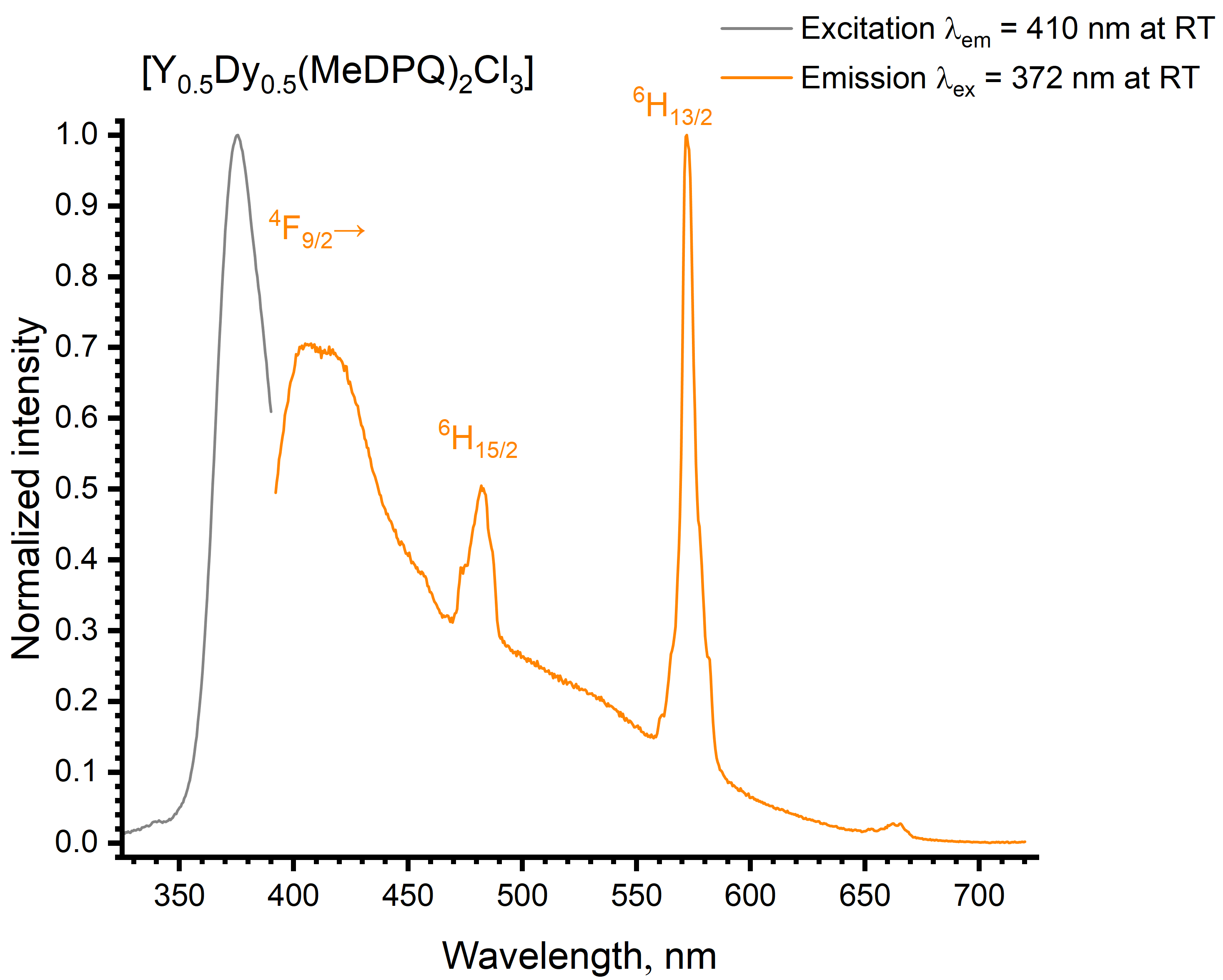


**Figure S15**. Normalized photoluminescence spectra of the solid-state sample [Y_0.5_Dy_0.5_(MeDPQ)_2_Cl_3_] at room temperature. Dy^3+^-based *f*–*f* transitions are labelled according to the energy levels.


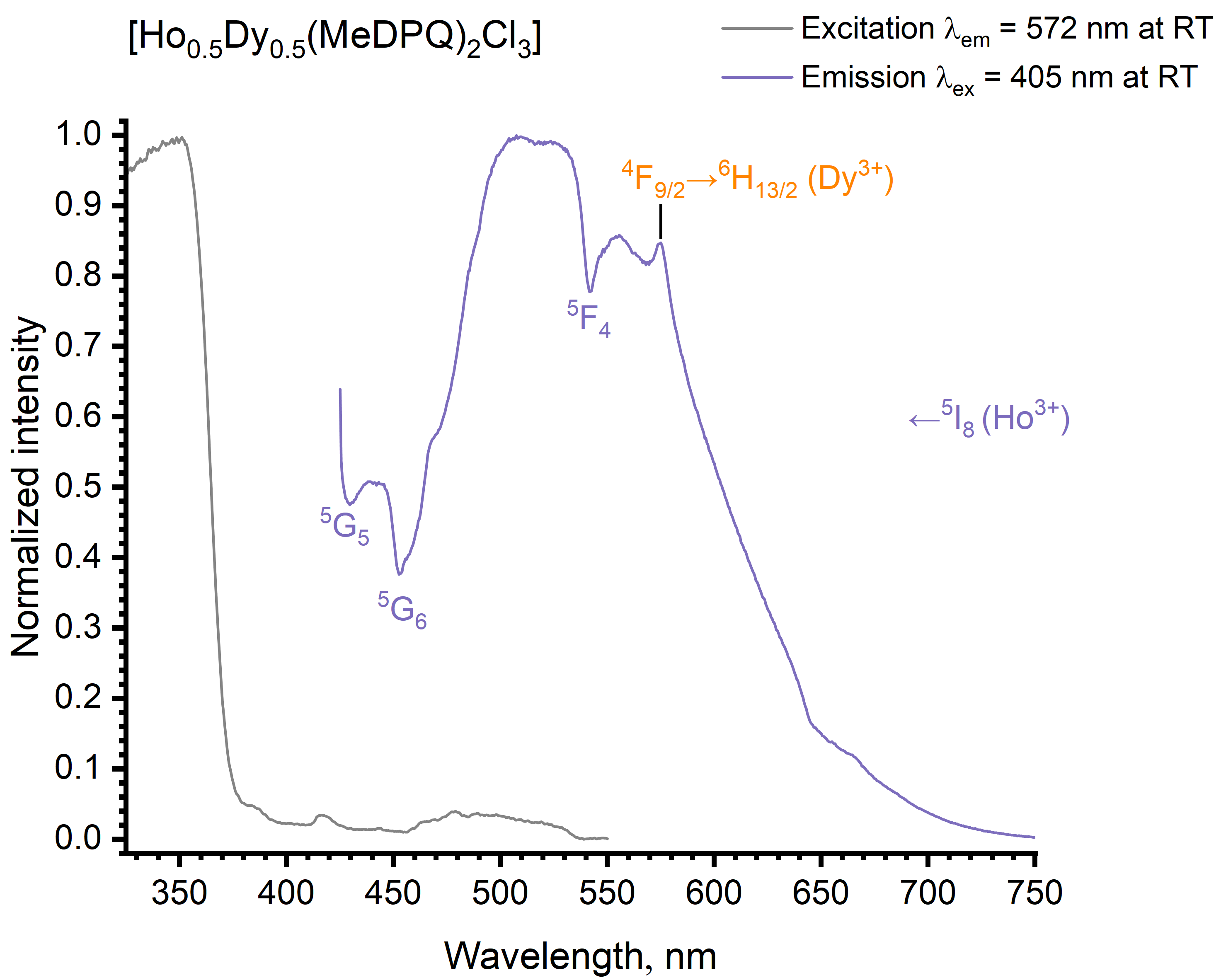


**Figure S16**. Normalized photoluminescence spectra of the solid-state sample [Ho_0.5_Dy_0.5_(MeDPQ)_2_Cl_3_] at room temperature. Dy^3+^-based *f*–*f* emission and Ho^3+^-based re-absorption of photons are labelled according to the energy levels.


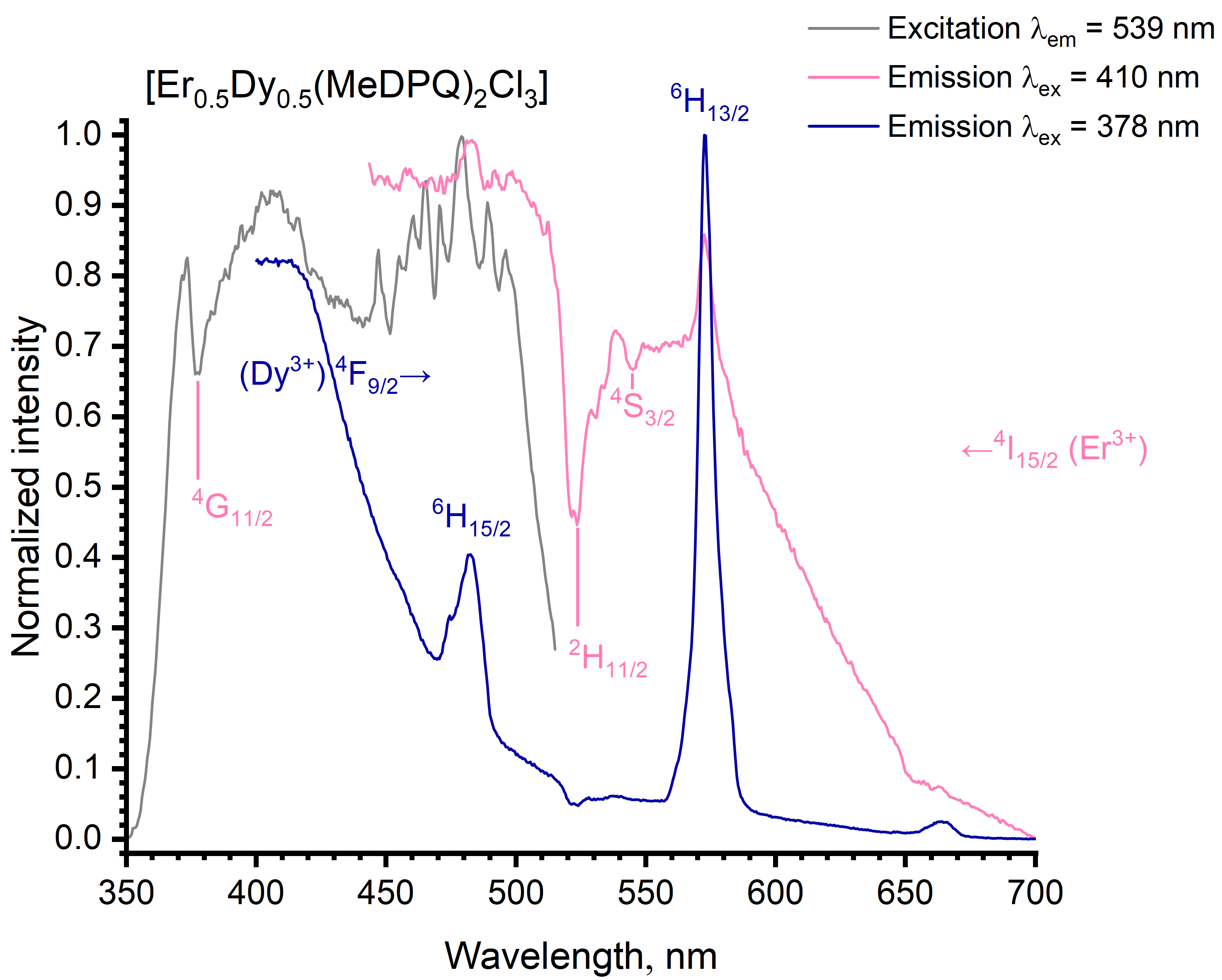


**Figure S17**. Normalized photoluminescence spectra of the solid-state sample [Er_0.5_Dy_0.5_(MeDPQ)_2_Cl_3_] at room temperature. Dy^3+^-based *f*–*f* emission and Er^3+^-based absorption of photons are labelled according to the energy levels.

Diffuse Reflectance Spectroscopy


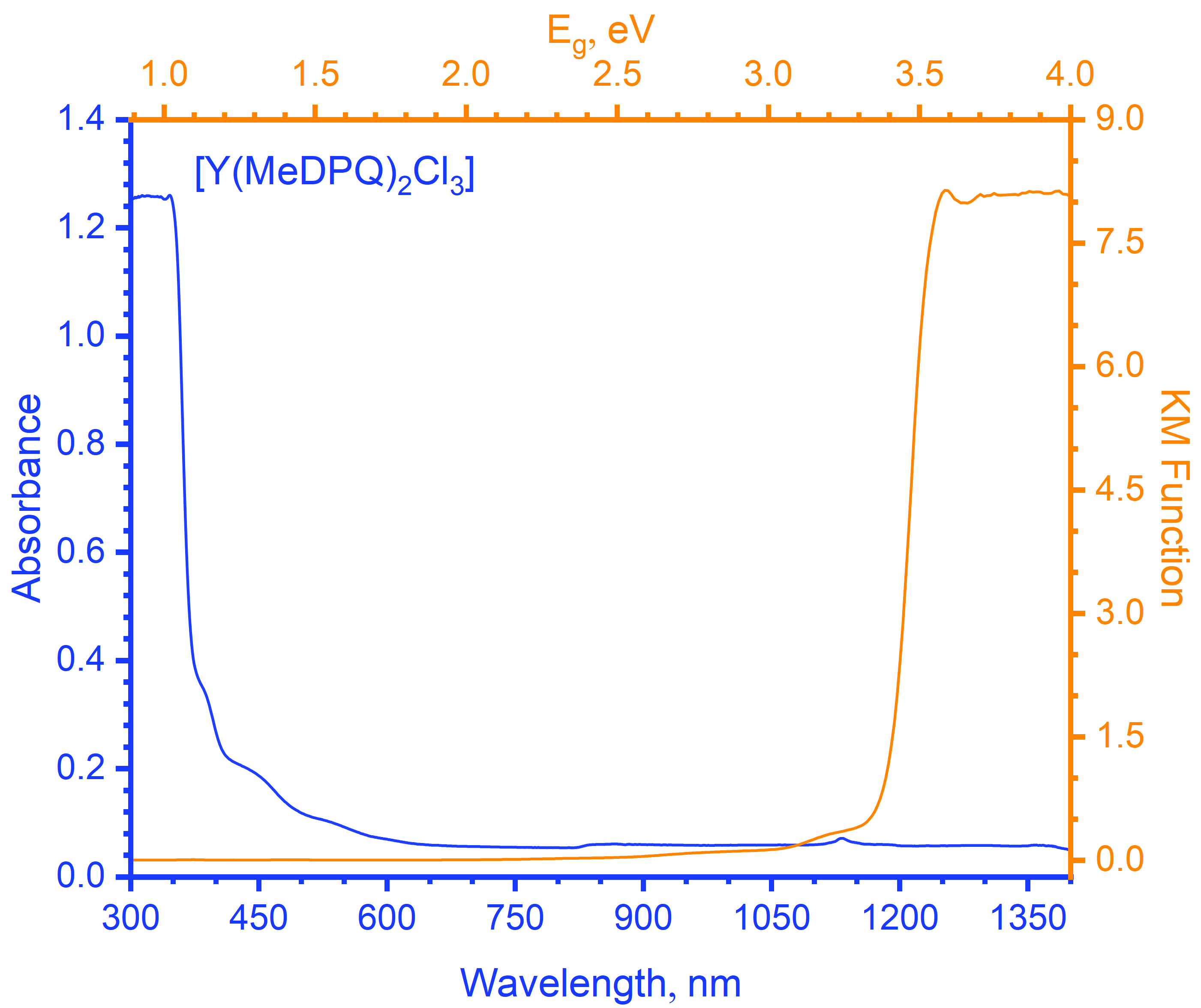


**Figure S18**. UV-Vis spectrum (blue) and Kubelka-Munk plot (orange) of the solid-state compound [Y(MeDPQ)_2_Cl_3_] at room temperature.


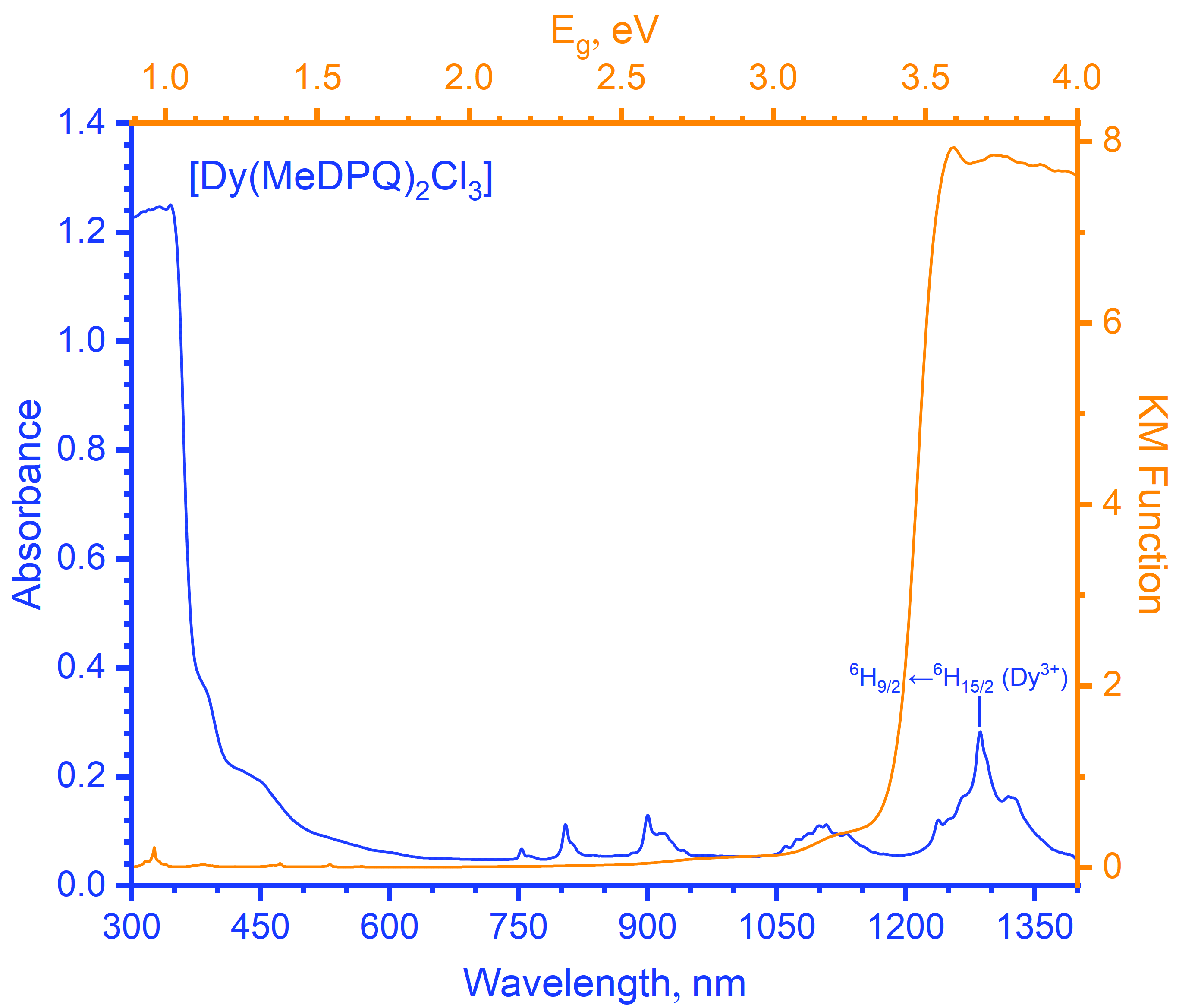


**Figure S19**. UV-Vis spectrum (blue) and Kubelka-Munk plot (orange) of the solid-state compound [Dy(MeDPQ)_2_Cl_3_] at room temperature. Ln^3+^-based *f–f* absorption is labelled according to the energy levels of respective ion.


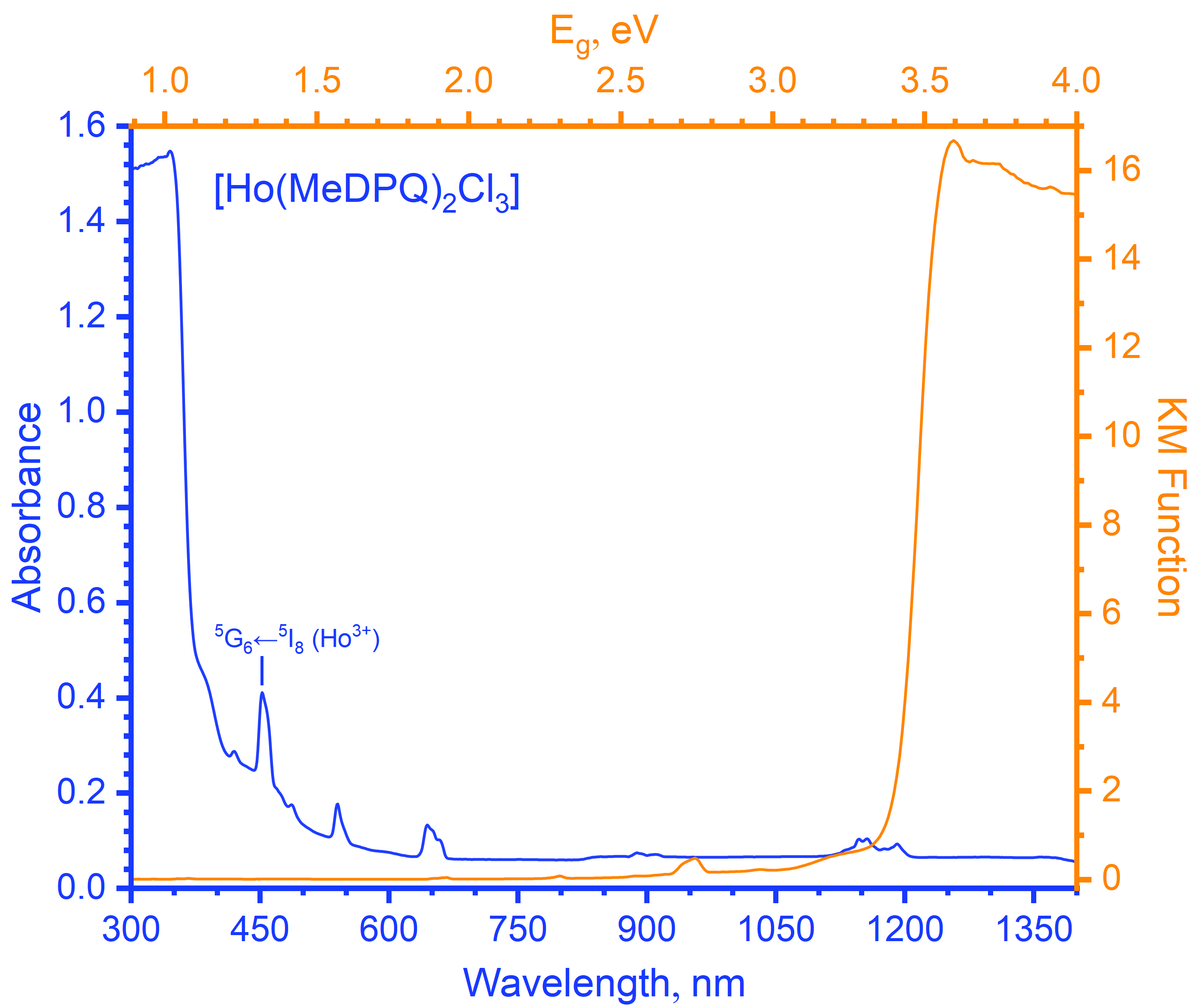


**Figure S20**. UV-Vis spectrum (blue) and Kubelka-Munk plot (orange) of the solid-state compound [Ho(MeDPQ)_2_Cl_3_] at room temperature. Ln^3+^-based *f–f* absorption is labelled according to the energy levels of respective ion.


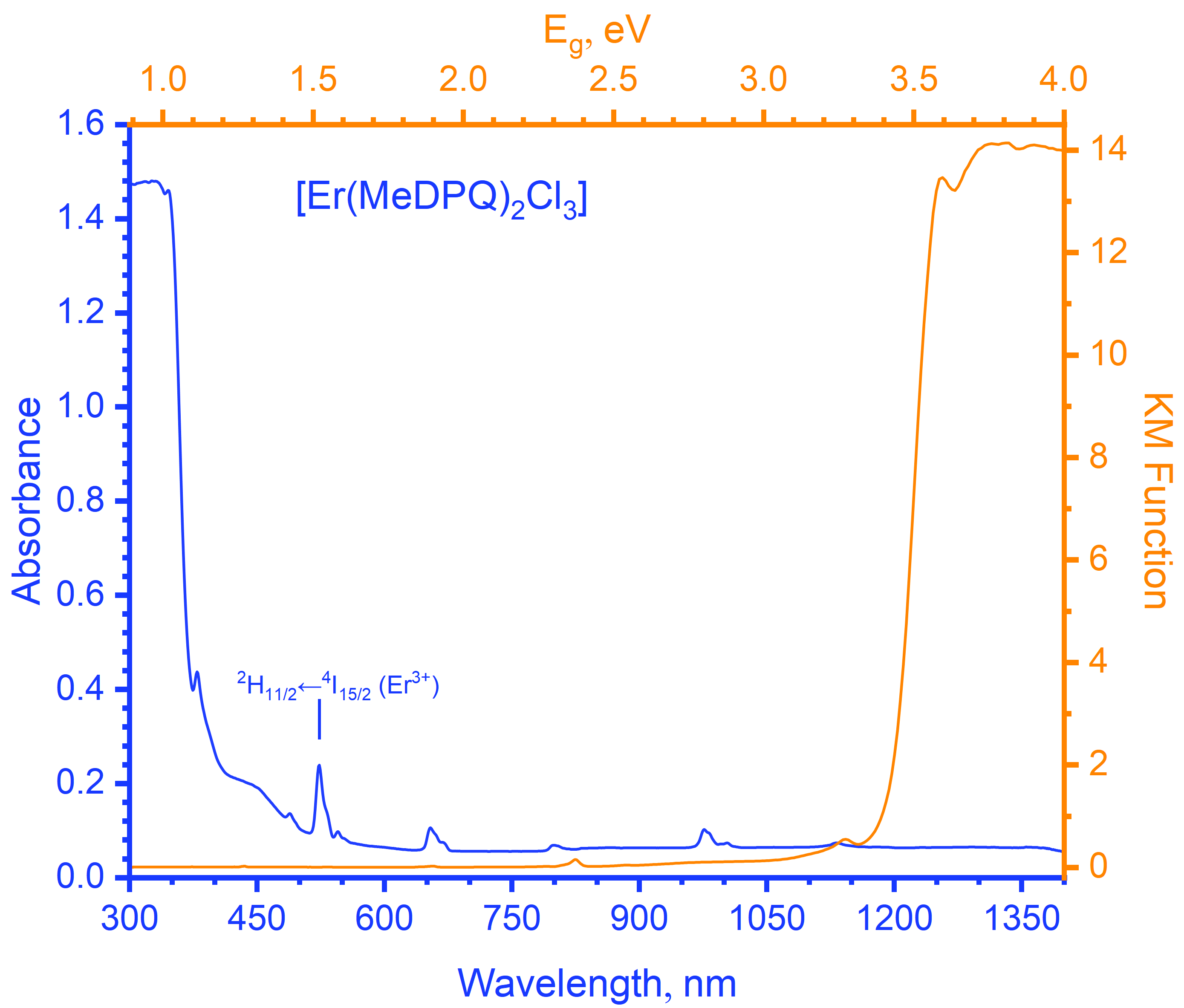


**Figure S21**. UV-Vis spectrum (blue) and Kubelka-Munk plot (orange) of the solid-state compound [Er(MeDPQ)_2_Cl_3_] at room temperature. Ln^3+^-based *f–f* absorption is labelled according to the energy levels of respective ion.


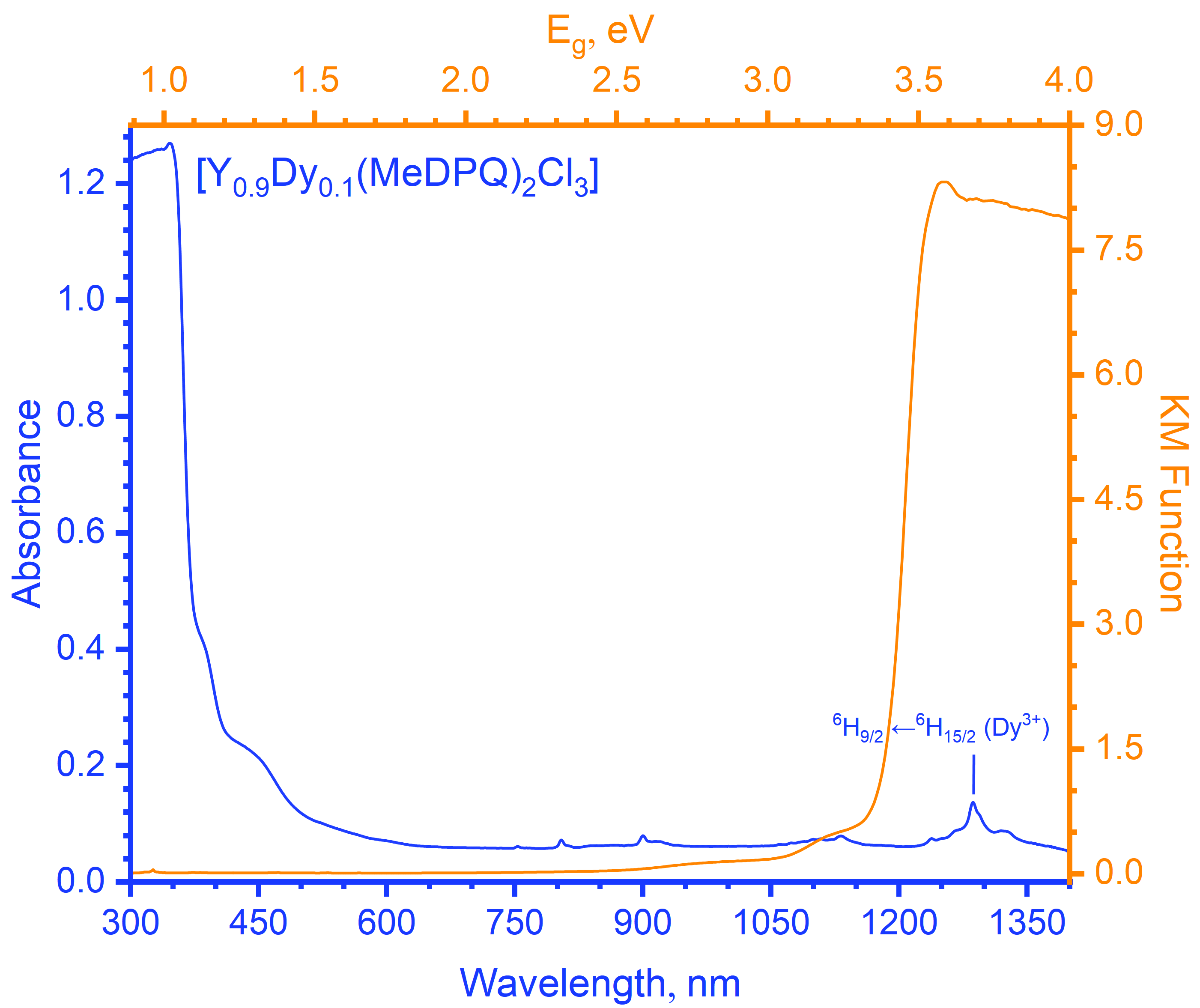


**Figure S22**. UV-Vis spectrum (blue) and Kubelka-Munk plot (orange) of the solid-state compound [Y_0.9_Dy_0.1_(MeDPQ)_2_Cl_3_] at room temperature. Ln^3+^-based *f–f* absorption is labelled according to the energy levels of respective ion.


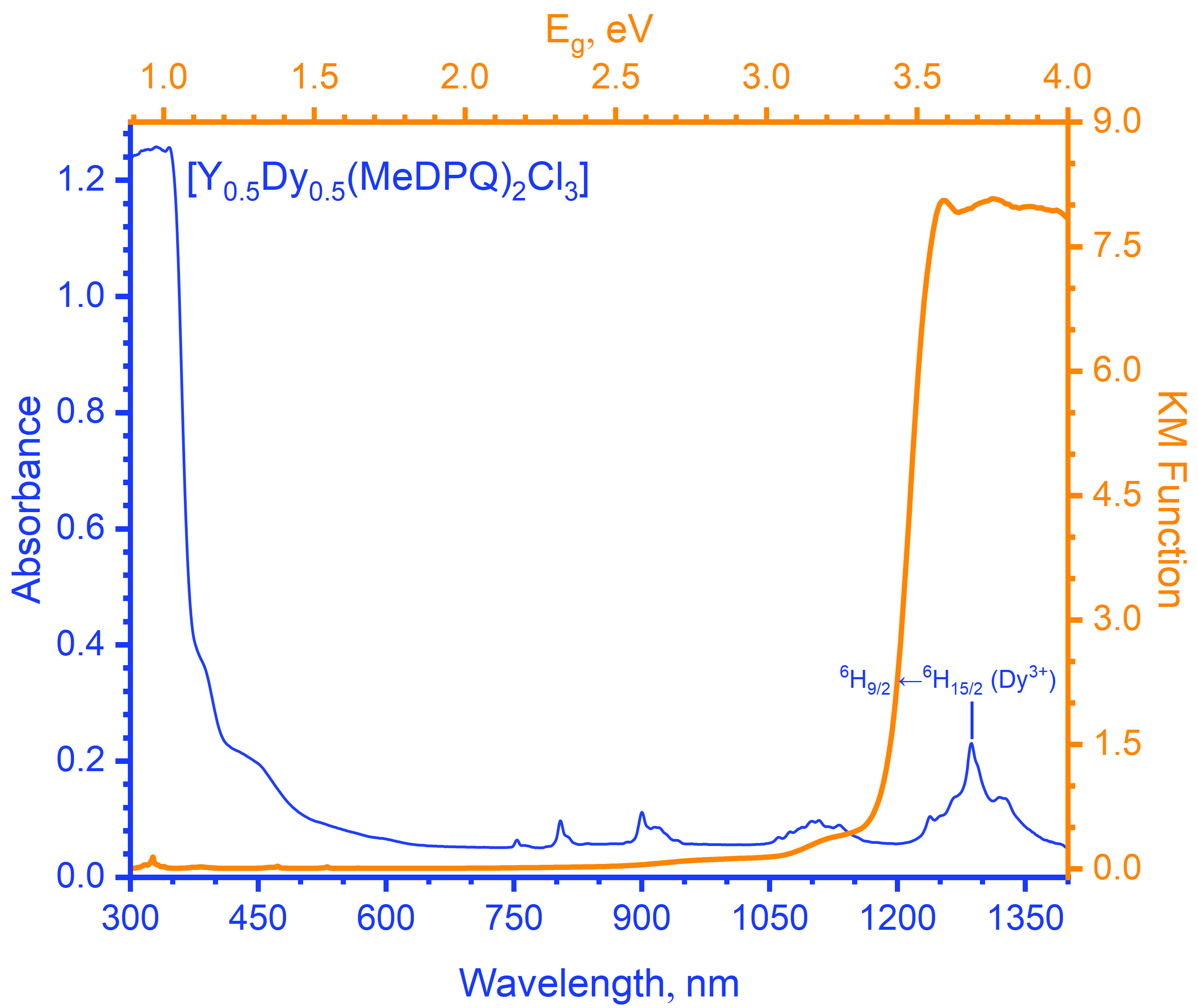


**Figure S23**. UV-Vis spectrum (blue) and Kubelka-Munk plot (orange) of the solid-state compound [Y_0.5_Dy_0.5_(MeDPQ)_2_Cl_3_] at room temperature. Ln^3+^-based *f–f* absorption is labelled according to the energy levels of respective ion.


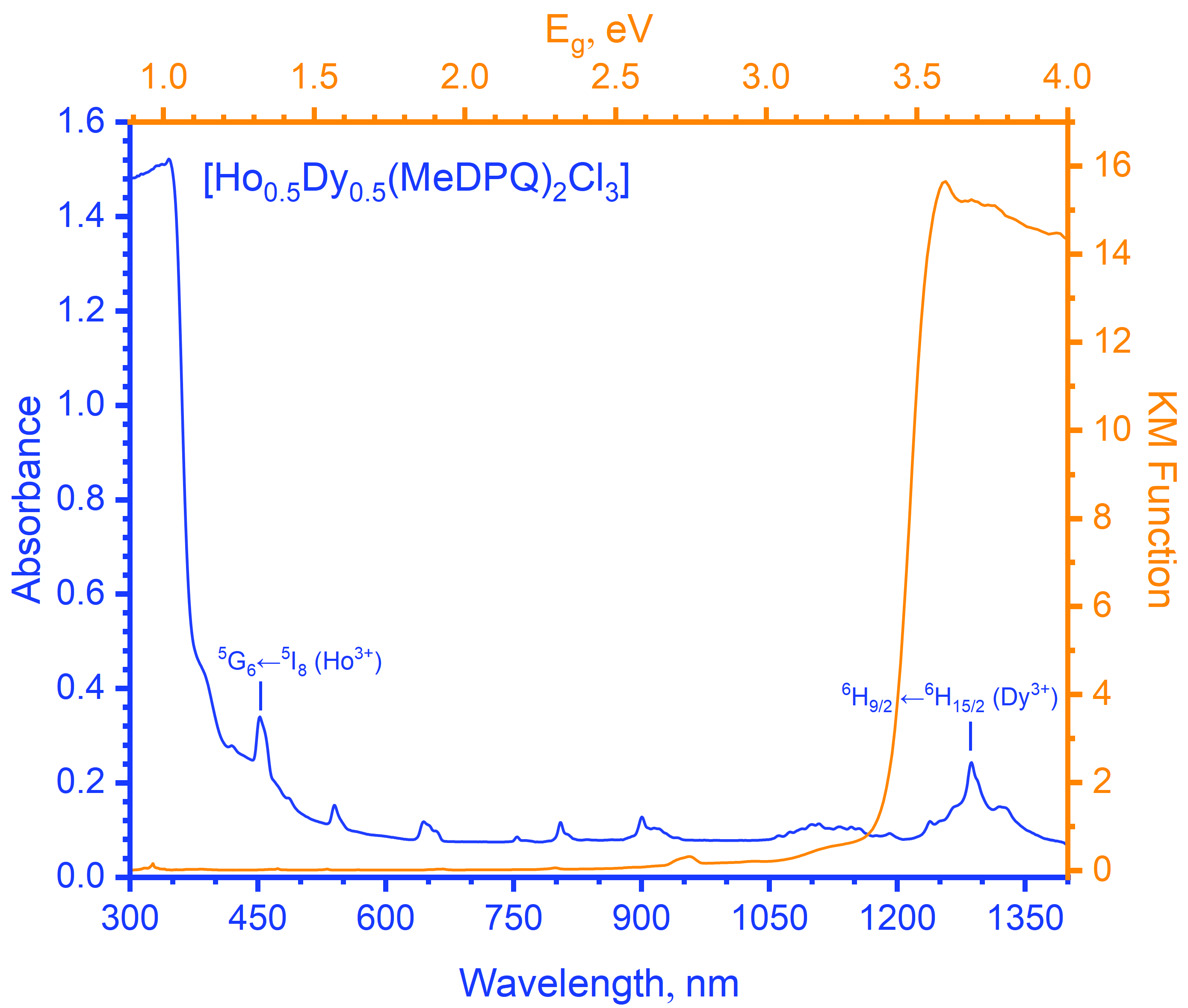


**Figure S24**. UV-Vis spectrum (blue) and Kubelka-Munk plot (orange) of the solid-state compound [Ho_0.5_Dy_0.5_(MeDPQ)_2_Cl_3_] at room temperature. Ln^3+^-based *f–f* absorption is labelled according to the energy levels of respective ion.


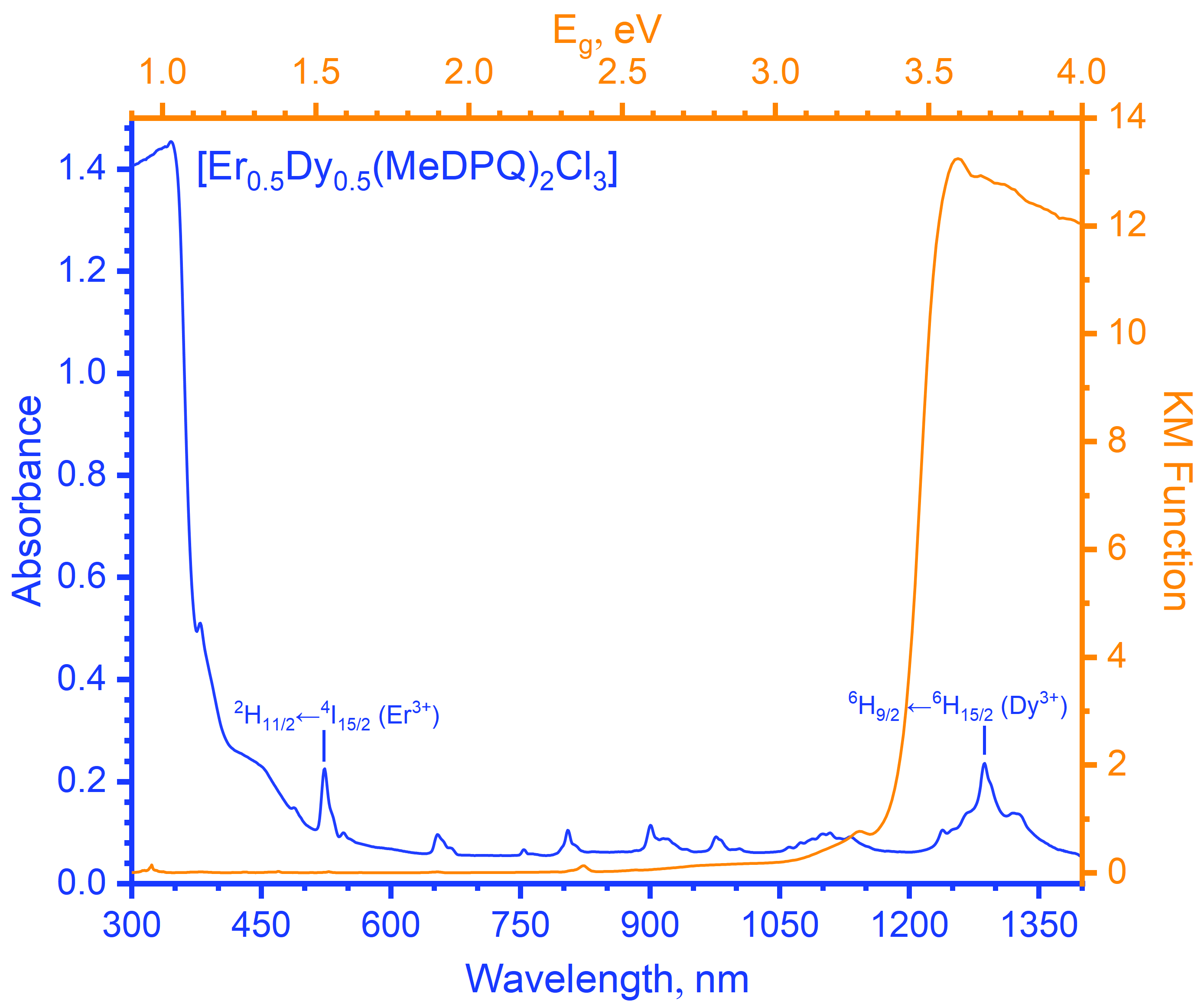


**Figure S25**. UV-Vis spectrum (blue) and Kubelka-Munk plot (orange) of the solid-state compound [Er_0.5_Dy_0.5_(MeDPQ)_2_Cl_3_] at room temperature. Ln^3+^-based *f–f* absorption is labelled according to the energy levels of respective ion.

Thermal Analysis


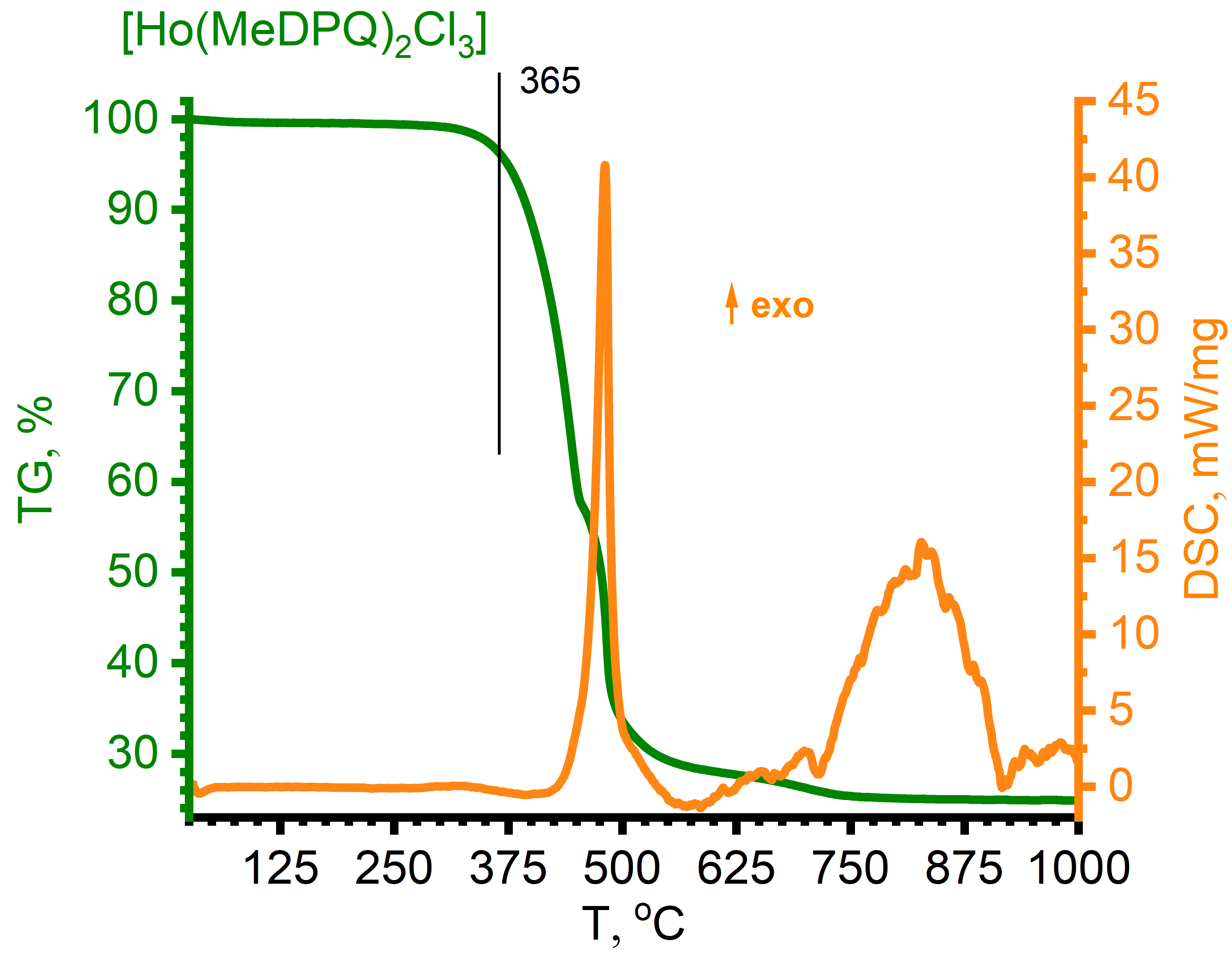


**Figure S26**. Simultaneous differential thermal analysis (DSC, orange) and thermogravimetry (TG, green) of the complex [Ho(MeDPQ)_2_Cl_3_]. The analysis was performed under synthetic air atmosphere.


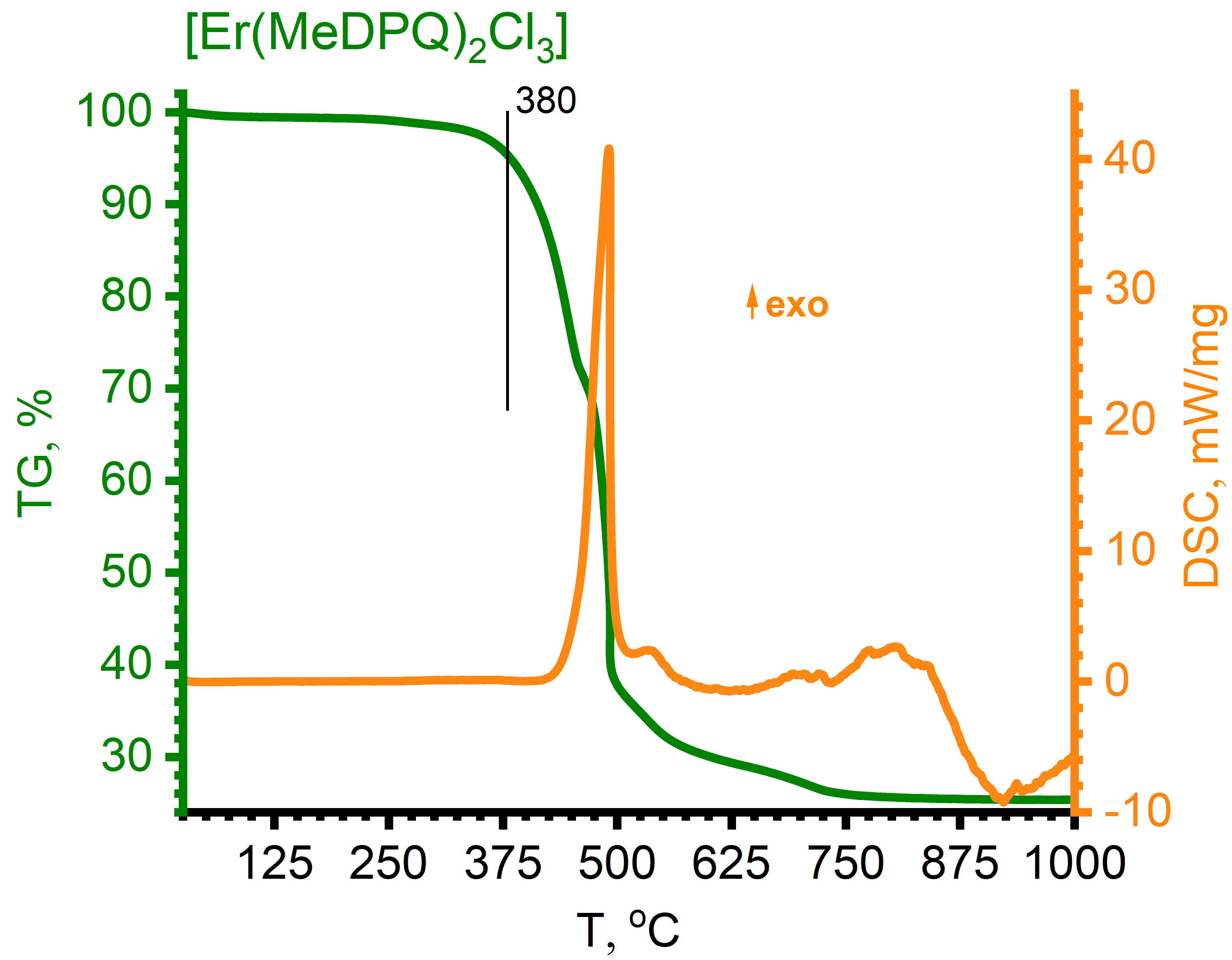


**Figure S27**. Simultaneous differential thermal analysis (DSC, orange) and thermogravimetry (TG, green) of the complex [Er(MeDPQ)_2_Cl_3_]. The analysis was performed under synthetic air atmosphere.


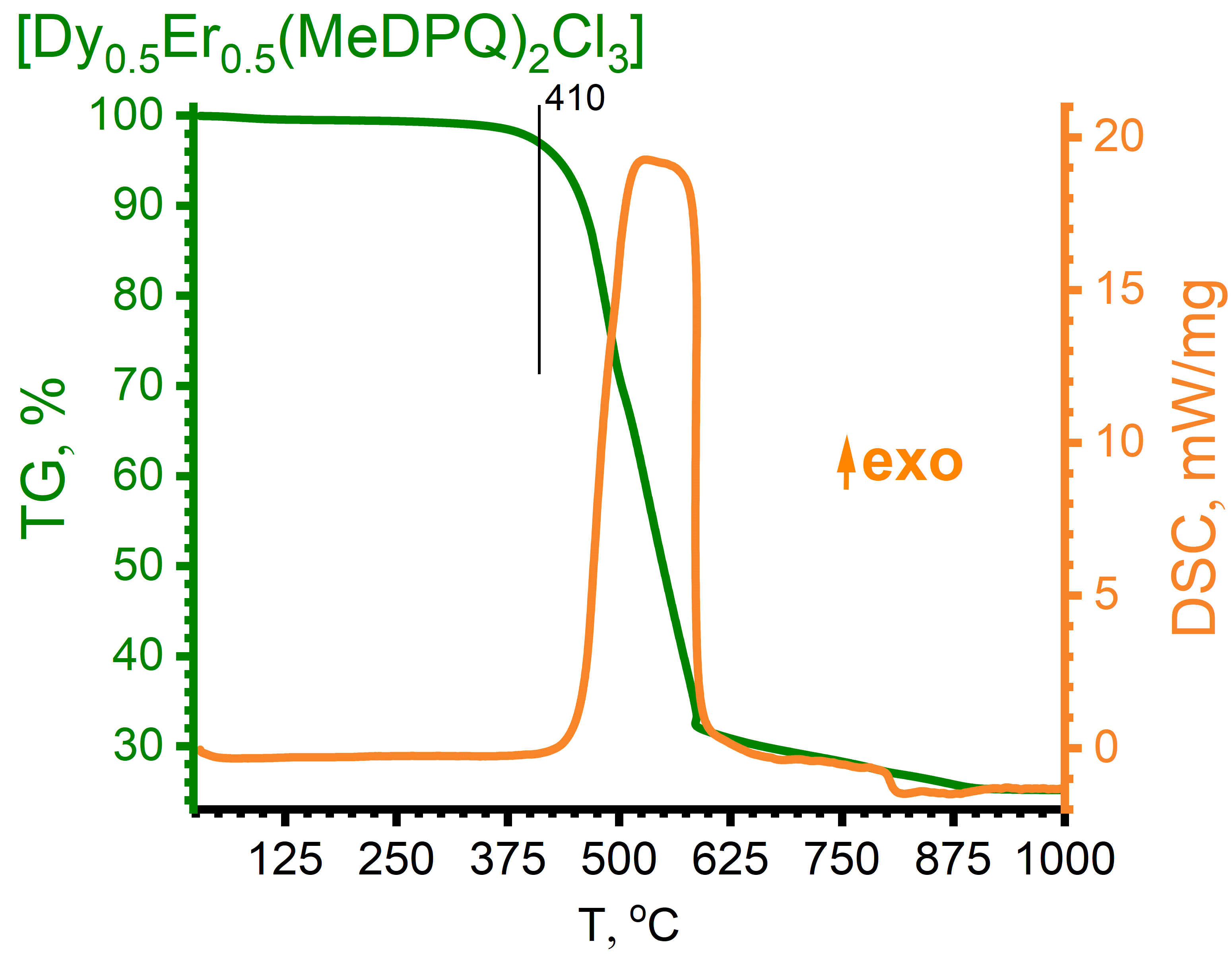


**Figure S28**. Simultaneous differential thermal analysis (DSC, orange) and thermogravimetry (TG, green) of the complex [Dy_0.5_Er_0.5_(MeDPQ)_2_Cl_3_]. The analysis was performed under synthetic air atmosphere.
